# A model predictive control-based energy management strategy for grid-connected nanogrids

**DOI:** 10.1038/s41598-026-68560-0

**Published:** 2026-09-06

**Authors:** Mohamed Selmy, A. Abdulnabi, A. A. Ali, E. M. Saied, W. T. Elsayed

**Affiliations:** 1 Department of Electrical Engineering, Faculty of Engineering at Shoubra, Benha University, Benha, Egypt; 2 Innovation University, Cairo, Egypt; 3 Department of Electrical Engineering, Faculty of Engineering, Helwan University, Cairo, Egypt

**Keywords:** Energy management strategy, Nanogrids, Goat optimization algorithm, Model predictive control, Demand side management, Energy science and technology, Engineering

## Abstract

Nanogrids have emerged as a crucial solution for contemporary residential power systems, providing a robust framework for integrating distributed energy resources (DERs), comprising solar photovoltaics (PVs), wind turbines (WTs), energy storage systems (ESSs), and diesel generators (DGs). However, the uncertainty of consumer demand and the inherent intermittency of renewable energy present significant challenges to maintaining stability, power quality, and cost-effective operation. This study proposes an effective two-level energy management strategy (EMS) to reduce operating costs, considering system limitations and uncertainties in energy demand, grid costs, and renewable energy sources. In the first level, load demand forecasts, renewable energy forecasts, and grid tariffs are used to schedule generation units and storage systems one day ahead of time. The resulting constrained nonlinear optimization problem is solved using the Goat Optimization Algorithm (GOA), a bio-inspired metaheuristic optimization algorithm. At the second level of the proposed EMS, a Model Predictive Control (MPC) strategy repeatedly solves a receding-horizon optimization problem to update the power set-points of the available energy resources in real time, addressing forecast inaccuracies in solar irradiation, wind speed, and demand. The proposed GOA-MPC-EMS is assessed through two test case scenarios: individual nanogrid operation and an integrated multi-nanogrid cluster within a grid-connected environment. The results indicate that the proposed GOA-MPC-EMS achieves cost-effective and robust operation for a grid-tied nanogrid under uncertainties in electricity prices, weather conditions, and load demand. To evaluate the effectiveness of the GOA for the optimal day-ahead scheduling of diesel generators and batteries, a comparison with three other optimization techniques, namely Dream Optimization Algorithm (DOA), Harris Hawks Optimization (HHO), and Particle Swarm Optimization (PSO), is conducted. According to simulation results, the day-ahead operating cost is reduced by approximately $23.85 with GOA, $22.83 with DOA, $18.30 with HHO, and $16.88 with PSO compared with the scenario without demand-side management (DSM), corresponding to cost savings of approximately 8.16%, 7.81%, 6.26%, and 5.78%, respectively. Meanwhile, the real-time EMS achieves additional daily savings of $16.39, corresponding to a further reduction in operating cost of approximately 6.11%.

## Introduction

Adoption of renewable energy has increased significantly in the last two decades due to the need to switch to more sustainable energy sources and minimize the negative environmental effects of power produced from fossil fuels^1^. To facilitate this shift, governments and the power industry have been progressively promoting decentralized generation through the deployment of Nanogrids (NGs). NGs are small microgrids, often designed to serve individual buildings, characterized by lower power capacity and simpler architectural complexity compared to microgrids^2^. A nanogrid (NG) integrates Distributed Energy Resources (DERs), such as Wind Turbines (WTs), Photovoltaic (PV) systems, Energy Storage Systems (ESSs), and Diesel Generators (DGs), with local electrical loads^3^.

The NG operates in both modes: islanded mode and grid-tied mode^4^. In grid-tied mode, the NG maintains the balance between electricity supply and demand by exchanging power with the utility grid. By reducing the physical distance between the energy source and the demand, NG installation reduces dependency on the main grid and, as a result, transmission losses^2^.

An effective energy management strategy (EMS) is required to schedule DERs and utilize current and historical data to manage controllable loads and facilitate power transfer with the utility while adhering to different technical constraints^5^. The EMS provides reference profiles for the controllers to govern power flow among the NGs in accordance with predefined objectives, e.g., reducing daily operating expenses^6,7^. To reduce overall energy consumption costs, controllable loads are deliberately shifted from high-priced to low-priced time intervals, effectively implementing the Demand Side Management (DSM) principle^8^. Renewable energy sources, including the most widely used ones such as WT and PV, are intermittent, producing power that fluctuates with environmental conditions. Furthermore, coordination between demand and supply, battery discharging and charging, power balancing, and keeping the battery state of charge (SOC) within acceptable limits—all these factors require a robust EMS to enable them. If this EMS is properly developed, the NG will be robust and cost-efficient.

Previous research on energy management in nanogrid systems has predominantly focused on improving power reliability using a multi-agent artificial intelligence architecture^9^, developing feeder reconfiguration strategies for networks with distributed generation units and electric vehicles (EVs)^10^, and integrating EV charging stations into distribution feeder reconfiguration to enhance operational performance^11^, improving energy trading performance using Internet of Things (IoT)-orchestrated frameworks^12^, addressing demand-side management issues through demand response programs (DRPs) to effectively manage renewable energy generation and load fluctuations^13^, and integrating energy storage units, such as community supercapacitors (SCs) and battery energy storage systems , to enhance system flexibility and operational reliability^14^, and using techniques for cost-sensitive scheduling^15^. A comprehensive review is necessary to critically evaluate the methodological frameworks, modeling approaches, and uncertainty handling constraints associated with optimization-based energy management solutions.

In^16^, an optimization-based EMS for a grid-connected NG was developed to minimize costs while maintaining reliable operation. In^17^, a rule-based EMS for an EV charging station NG was proposed to enhance operational reliability and economic performance under variable charging conditions. In^18^, an EMS implementation approach for droop-controlled residential DC NGs was proposed to ensure stable operation and efficient power sharing. Nevertheless, these studies’ EMS frameworks did not incorporate demand-side management.

In^19^, a multilayer DC NG architecture using vehicle-to-grid (V2G) technique was proposed to optimize resource utilization, reduce cost, and enhance power dispatch coordination. In^20^, a PSO-based EMS integrating supply and DSM was developed to increase energy efficiency and save costs. In^21^, a hierarchical EMS using model predictive control and reinforcement learning was proposed to maximize energy management and improve operational efficiency in building clusters. However, the aforementioned studies failed to account for inherent uncertainty in weather patterns, load predictability, and grid tariff within the proposed EMSs.

In^22^, a DSM-based EMS was recommended to improve reliability and reduce cost in a PV-integrated NG. In^23^, a day-ahead scheduling EMS incorporating demand response and renewable energy was developed to enhance grid operation flexibility and optimize power scheduling. In^24^, a model predictive control technique for optimal scheduling of residential EMSs was proposed to improve operational decision-making under dynamic energy conditions. However, real-time operation was not considered in these studies.

In^5^, to enable optimal economic operation, a real-time optimum scheduling approach for grid-connected microgrids that incorporates uncertainty mitigation and load management was proposed. In^25^, to guarantee optimal economic performance, a two-layer EMS enables grid-connected multi-NGs to operate optimally in real time was investigated. In^26^, a multi-layer optimum EMS for grid-tied microgrids that considers weather and load uncertainty was proposed to illustrate optimal economic operation. These studies adopted 1-h interval scheduling in real-time operation and lacked rigorous statistical validation to achieve the robustness of the optimization results.

NGs pose intricate challenges arising from the nonlinear behaviour of grid components, the variety of DERs, and the random nature of load demand. Different solution techniques have been suggested to tackle the nonlinear issues in NGs. Conventional deterministic techniques, specifically mixed-integer linear programming (MILP), have been frequently applied in previous research^24^. Although MILP-based methods offer structured modeling and computational efficiency under linear assumptions, their reliance on linearization and precise system modeling may limit their effectiveness in handling highly nonlinear, large-scale, and uncertain optimization problems. To address these challenges, various evolutionary and metaheuristic algorithms was proposed such as PSO^6^, Honey Badger Algorithm (HBA)^5^, Pelican Optimization Algorithm (POA)^27^, Artificial Intelligent (AI)^9^, and Reinforcement Learning (RL)^21^, which have proven to be highly effective in handling complex search spaces, nonlinearity, and uncertainty without the need for gradient information.

Despite the significant progress reported in the literature, several critical research gaps remain.

- DSM is neglected in most NG EMSs.
- Many current models do not take into consideration the stochastic nature of load power, grid prices, and weather conditions, which all substantially affect renewable energy during real-time operation.
- Existing studies show insufficient adoption of advanced and recent metaheuristic techniques for solving the problem, while rigorous statistical analysis is frequently overlooked, even though it is crucial for ensuring the robustness, consistency, and reliability of the proposed approaches.

Table 1 summarizes representative previous studies on NG energy management relevant to the proposed work.

**Table 1:** Comparative summary of previous studies on NG energy management.

References	DSM	Uncertainty	Day-ahead	Real-time	Statistical validation
^16^	✗	✗	✗	✓	✗
^17^	✗	✓	✗	✗	Limited
^19^	✗	✗	✓	Partial	✗
^20^	✓	✗	✓	✗	✗
^21^	Partial	Limited	✗	✓	Partial
^23^	✓	Partial	✓	✗	✓
^24^	✓	✗	✓	✗	✗
^25^	✓	✓	✓	✓	✗
This work	✓	✓	✓	✓	✓

**Fig. 1:**
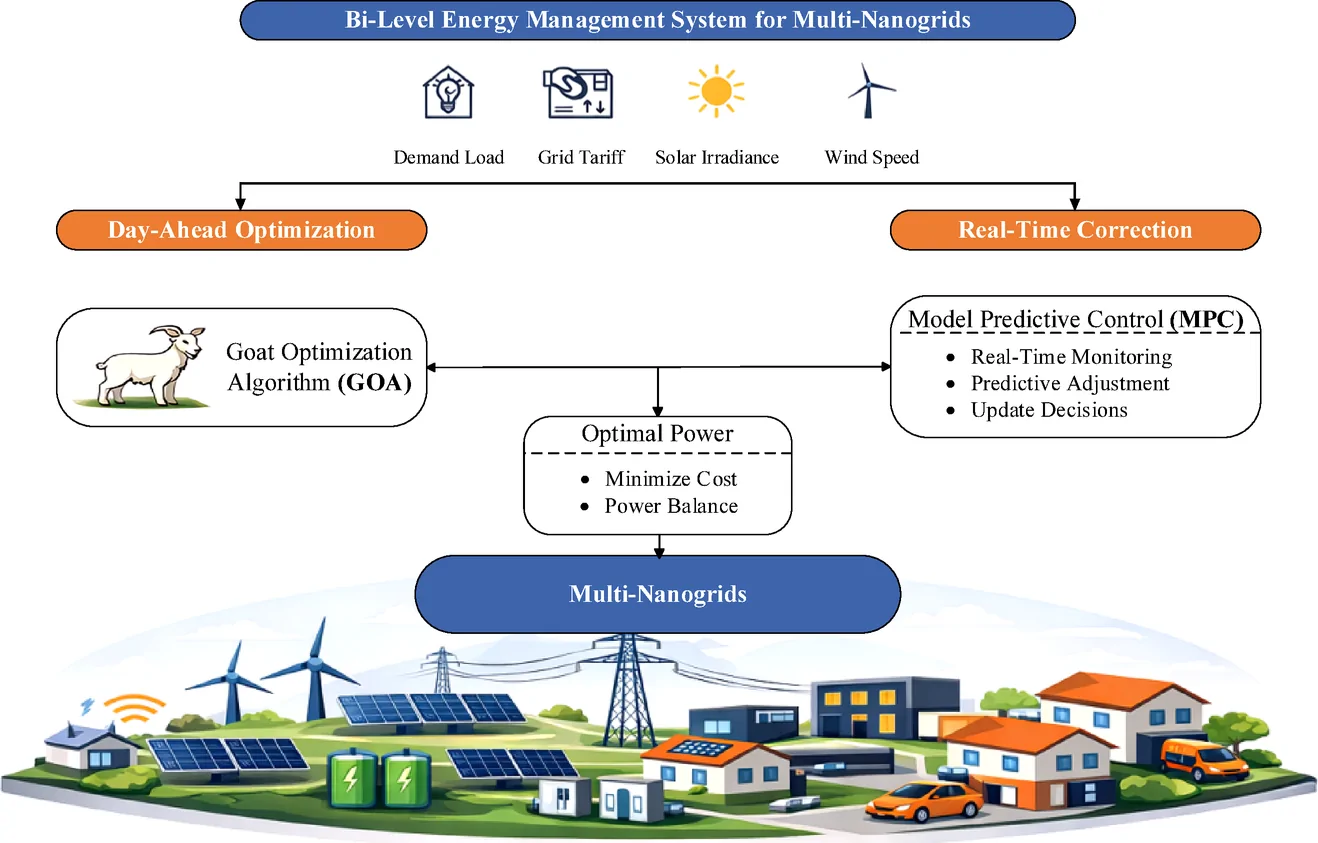
Graphical representation of the proposed two-level EMS framework (created using Microsoft Visio, Microsoft 365 Version 2607; https://www.microsoft.com/visio).

To overcome these gaps, this study recommends a two-level energy management strategy that minimizes the daily operational costs of grid-connected NGs through optimal coordination of their energy sources. The Goat Optimization Algorithm (GOA) is employed to efficiently solve the resulting complex nonlinear optimization problem, and its performance is benchmarked against the Dream Optimization Algorithm (DOA), Harris Hawks Optimization (HHO), and Particle Swarm Optimization (PSO) in day-ahead scheduling, while Model Predictive Control (MPC) is adopted for real-time operation. Figure 1 displays the general architecture of the proposed EMS.

The following are the main contributions of this work:

- Establishing an integrated EMS for multi-NGs that combines ANN-based forecasting, DSM through an intelligent load-shifting strategy, and day-ahead scheduling to minimize daily operating costs.
- Incorporating an MPC strategy into the proposed EMS to address the uncertainty associated with the forecasted weather conditions, electricity tariffs, and demand fluctuations, enabling continuous adjustment of distributed energy resources.
- Proposing the Goat Optimization Algorithm (GOA) for solving the optimization model. The performance is evaluated against PSO, HHO, and DOA, validated by thorough statistical validation to show resilience, convergence consistency, and improved performance, including several independent runs and Wilcoxon signed-rank tests.

## Proposed system structure

**Fig. 2:**
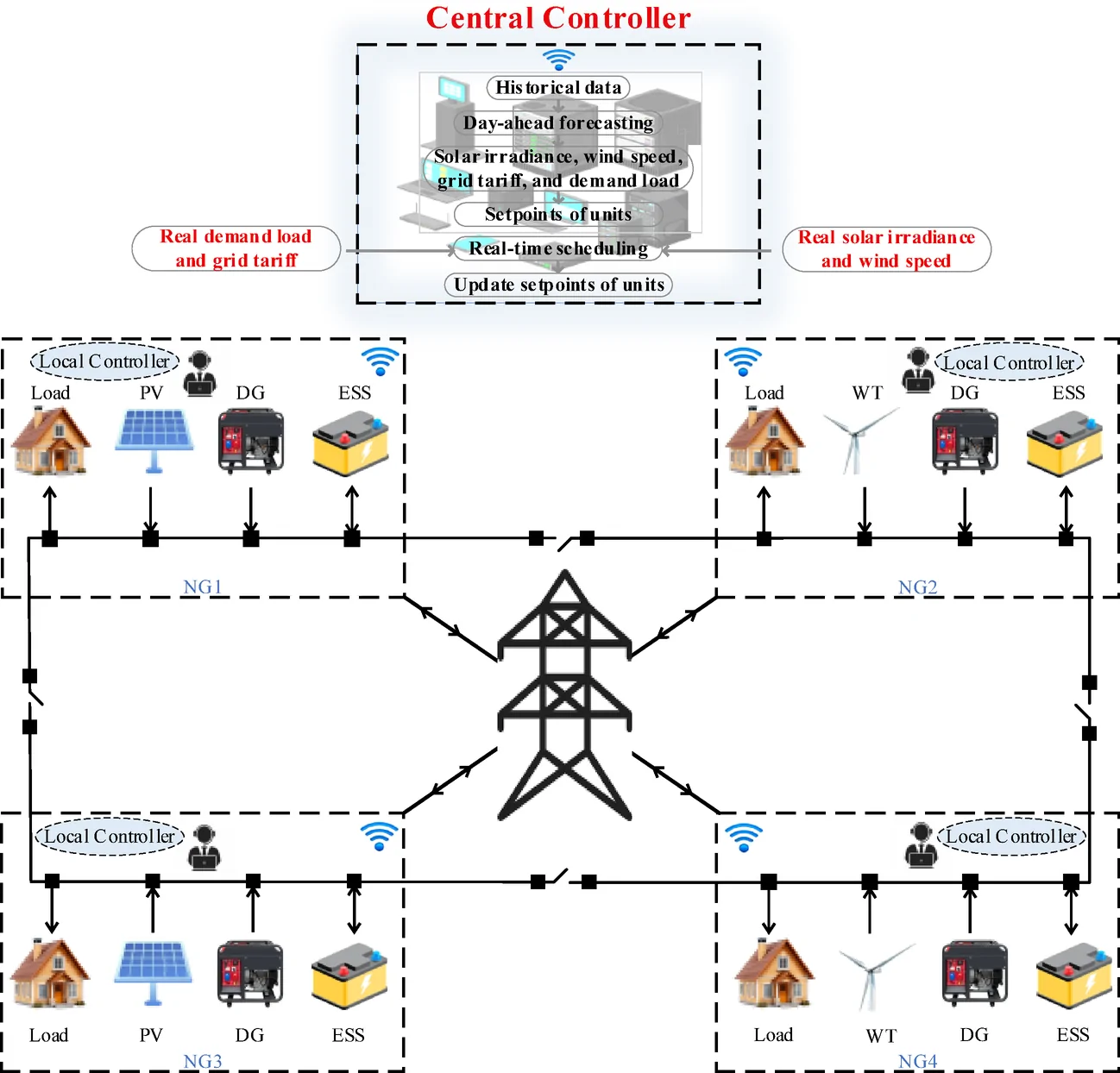
System structure (created using Microsoft Visio, Microsoft 365 Version 2607; https://www.microsoft.com/visio).

The grid-tied NGs system used in this study comprises four NGs (NG1-NG4), as depicted in Fig. 2. These four units are interconnected through a common DC bus and connected to the utility via an AC bus. In this configuration, NG1 and NG3 are outfitted with a 20-kWh battery, an 8-kW diesel generator, and five 4-kW PV modules. On the other hand, NG2 and NG4 consist of a 20-kWh battery, an 8-kW diesel generator, and four 5-kW wind turbines. Each source in the NGs is managed by a local controller. For example, the battery can be operated in both charging and discharging modes, whereas the PV or WT systems are capable of operating either in maximum power tracking mode or in power management mode. The central controller seeks to keep system stability and enhance EMS performance across the four NGs. It communicates via Wi-Fi with all sources and loads within the NG, enabling real-time decision-making for efficient and economical operation. The centralized EMS ensures fair energy sharing by considering individual operating and battery degradation costs for each nanogrid. Individual limits on battery SOC, charge/discharge power, renewable generation, and tie-line capacity are enforced. Thus, energy sharing occurs only when economically beneficial, avoiding excessive dependence on any single nanogrid. In the following section, the proposed EMS will be addressed in further detail. The appendix provides the system parameters^25,26^. All simulations were carried out using the MATLAB/Simulink platform.

### Wind turbine model

The generated power, *P*_*w*_, of a wind system at time *t*, composed of a number of wind turbines, *N*_*w*_, is determined by^5^:

1 $$ P_{wt} (t) = N_{w} \times \eta_{conv} \times \left\{ {\begin{array}{*{20}l} 0 \hfill & {{\mathrm{if}}\;v_{cf} < v(t) < v_{ci} } \hfill \\ {P_{w} \times \left( {\frac{{v(t) - v_{ci} }}{{v_{r} - v_{ci} }}} \right)^{3} } \hfill & {{\mathrm{if}}\;v_{ci} < v(t) < v_{r} } \hfill \\ {P_{w} } \hfill & {{\mathrm{if}}\;v_{r} < v(t) < v_{cf} } \hfill \\ \end{array} } \right. $$

where: (*v*_*cf*_, *v*_*ci*_) is the cut-off and cut-in wind speed, (*v*_*r*_, *v(t)*) is the rated and actual wind speed, and *η*_*conv*_ is the converter efficiency.

### Photovoltaic model

The generated power of the PV at time *t*, is determined by the cell temperature and the incident solar irradiation, as^25^:

2 $$ P_{pv} (t) = G(t) \times A \times \eta_{pv} \times (1 - \alpha_{pv} (T_{a} (t) - 25)) \times N_{pv} \times \eta_{conv} . $$

where: *G(t)* is the solar density, (*η*_*conv*_, *η*_*pv*_) is the converter and the PV efficiency*, A* is the area of the PV module, *α*_*pv*_ is the power coefficient of PV, *T*_*a*_*(t)* is the typical surrounding temperature, and *N*_*pv*_ is the PV module’s number of cells.

### Battery model

The power of the battery as a function of time *t* should be limited within the following lower and upper bounds:

3 $$ P_{B,\min } < P_{B} (t) < P_{B,\max } . $$

The battery’s stored energy and related SOC can be defined as^26^:

4 $$ E(t) = \left\{ {\begin{array}{*{20}l} {E(t - 1) - \frac{{\Delta T \times P_{B} (t)}}{{\eta_{dis} }}} \hfill & {{\mathrm{if}}\;P_{B} (t) > 0} \hfill \\ {E(t - 1) - \Delta T \times P_{B} (t) \times \eta_{ch} } \hfill & {{\mathrm{if}}\;P_{B} (t) < 0} \hfill \\ \end{array} } \right.. $$

where: (*η*_*dis*_, *η*_*ch*_) is the efficiency of the battery during discharging and charging operation, *ΔT* is the sampling time.

The battery state of charge can be outlined as:

5 $$ SOC(t) = \frac{E(t)}{{B_{capacity} }} $$

where *B*_*capacity*_ is the capacity of the battery.

The lower and upper boundaries should be used to limit the battery’s state of charge (SOC), as:

6 $$ SOC_{\min } < SOC(t) < SOC_{\max } $$

### Diesel generator model

The operational cost of the diesel generator can be defined as a quadratic formula of its generated power^26^:

7 $$ C_{dg} (t) = \alpha + \beta P_{dg} (t) + \gamma P_{dg} (t)^{2} $$

The diesel power delivery must fall within the maximum and minimum power boundaries, as follows:

8 $$ P_{dg,\min } < P_{dg} (t) < P_{dg,\max } $$

### Main grid model

The power transferred through the interconnecting line between the NGs and the main utility must not exceed the permitted limits, which are determined by stability and thermal factors:

9 $$ P_{g,\min } < P_{g} (t) < P_{g,\max } $$

The NGs overall active power balance is computed by:

10 $$ P_{g} (t) = P_{l} (t) - P_{pv} (t) - P_{wt} (t) - P_{dg} (t) - P_{B} (t) $$

where: *P*_*l*_*(t)* is the demand load of the NGs.

## Proposed energy management strategy

To meet the system requirements, the recommended EMS goals are to ensure optimal power exchange between diesel generators, batteries, and the utility, thereby satisfying load demand and reducing the operating cost of the NGs during the day, as defined in (11):

11 $$ Min\;C_{op} = \sum {\left[ {C_{dg} (t) + C_{g - sell} (t) + C_{g - buy} (t) + C_{batt} (t)} \right]} $$

where: *C*_*dg*_*(t)* is the diesel fuel cost ($), *C*_*g-sell*_*(t)* is the NGs sale price of electricity to the grid ($), *C*_*g-buy*_*(t)* is the NGs purchase price of electricity from the grid ($), and *C*_*batt*_*(t)* is the operating cost of the battery ($) at time *t*, which are calculated as^5^:

12 $$ C_{g - sell} (t) = P_{grid} (t) \times Tariff_{sell} (t),\;for\;P_{grid} (t) < 0 $$

13 $$ C_{g - buy} (t) = P_{grid} (t) \times Tariff_{buy} (t),\;for\;P_{grid} (t) > 0 $$

14 $$ C_{batt} (t) = \frac{{CC_{Bat} \times \Delta T}}{{N_{cycle} }} \times \left( {\frac{{P_{B,ch} (t) \times \eta_{ch} }}{2} + \frac{{P_{B,dis} (t) }}{{\eta_{dis} \times 2}}} \right) + C_{\deg } \times \left| {P_{B} (t)} \right| + C_{o - m} $$

where: *Tariff*_*sell*_*(t)*, *Tariff*_*buy*_*(t)* are the selling and buying energy tariffs, *CC*_*Bat*_ is the fixed cost of the battery, *C*_*o_m*,_ *C*_*dg*_ are the daily operating and maintenance cost and the degradation battery cost, and *N*_*cycle*_ is the battery’s life cycle number.

**Fig. 3:**
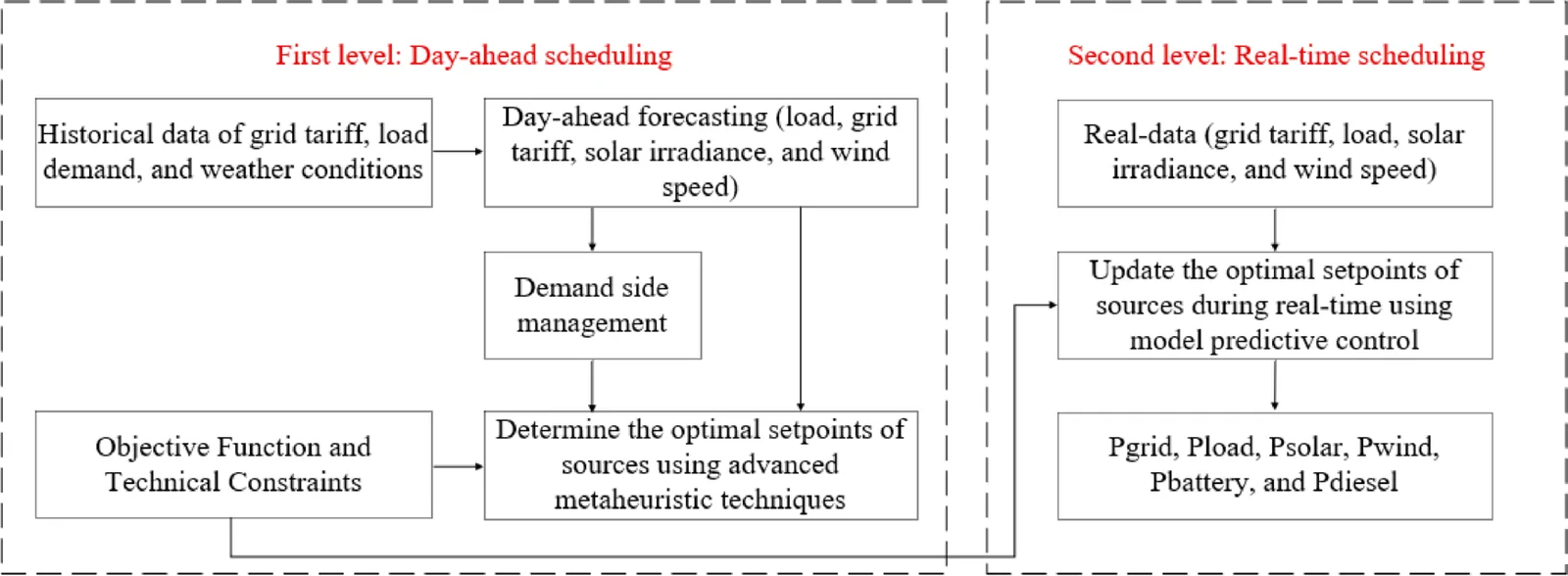
Proposed energy management system.

The proposed EMS works in two levels (day-ahead scheduling and real-time scheduling) to fulfill the load requirements of the NGs at minimal operational cost, using forecasted data for the next day and actual data. Figure 3 illustrates the flow chart of the recommended EMS.

### Level I: day‑ahead scheduling

This level consists of three phases: forecasting of electricity prices, solar irradiation, wind speed, and load demand in the first phase; DSM in the second phase; and scheduling of generation units in the third phase for upcoming day.

#### Day‑ahead forecasting (phase I)

This research applies short-term forecasting for demand load, grid tariff, and weather conditions. An Artificial neural network (ANN) is employed in this work for predicting the grid tariff, load demand, solar intensity, and wind speed for the upcoming day based on previous data. Additionally, this work examines the performance of three distinct forecasting models: the conventional linear regression (LR), support vector machine (SVM), and the proposed ANN-based forecasting^28,29^.

- Artificial neural networks (ANN)

**Fig. 4:**
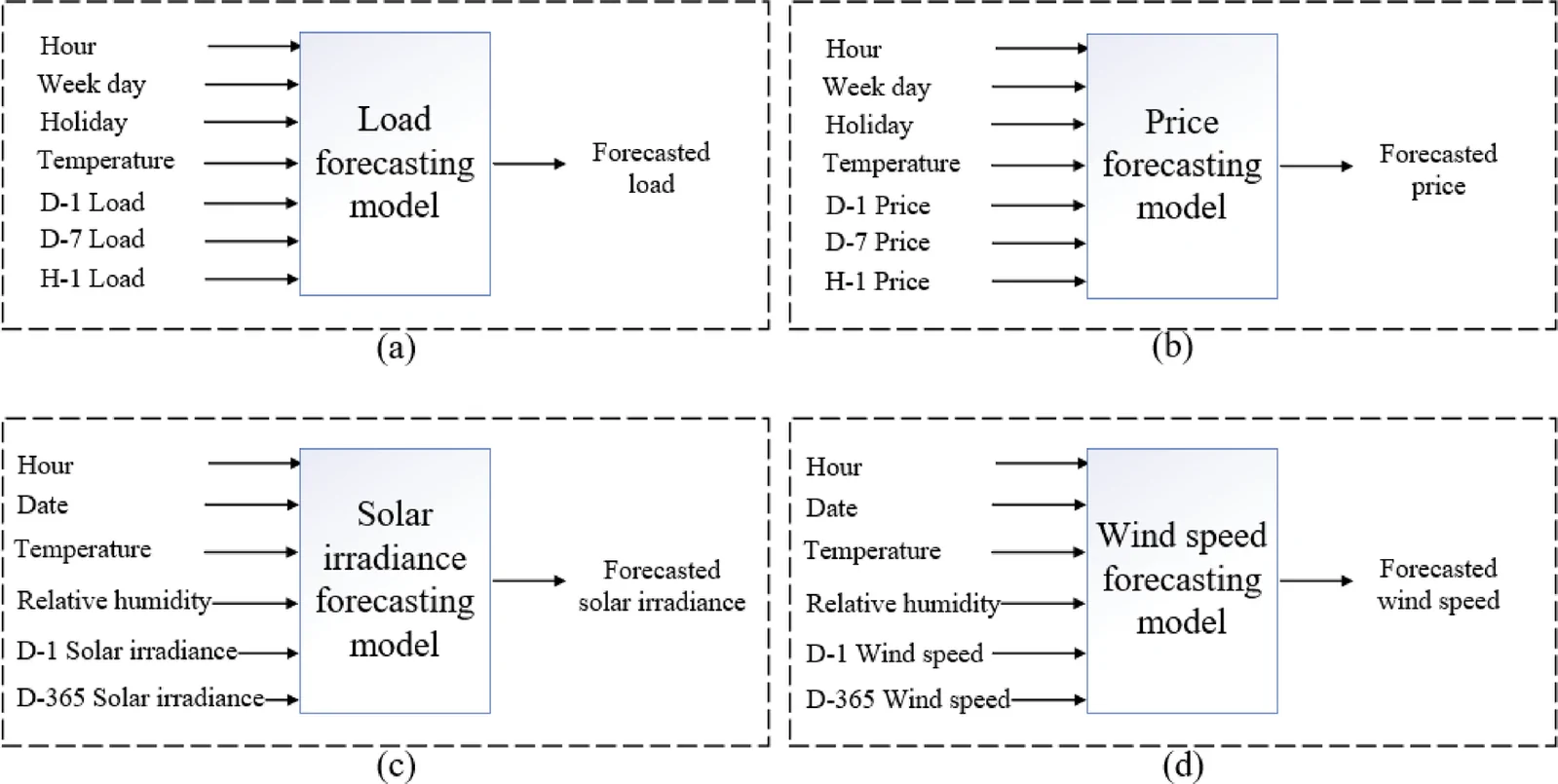
Model-based forecasting.

ANN is an instance of machine learning that models the structure of the human brain^17^. The neural network used for the forecasting model contains a hidden layer with 100 neurons, an input layer with seven neurons, and an output layer with one neuron, As can be observed from Fig. 4a, the load forecasting model uses seven variables as input data: hour, day, holiday indicator (one for a working day and zero for a holiday), temperature, D-1 load (the load from the day before), D-7 load (the load from the weak before), and H-1 load (the load of the before hour). The output of the model is the forecasted load for the upcoming day. As shown in Fig. 4b, the price forecasting model is similar to the load‑based forecasting model. Six variables formed the input data for the model-based solar irradiance forecasting model: hour, date, temperature, relative humidity, D-1 solar irradiance (the previous day’s solar irradiance), and D-365 solar irradiance (the previous year’s solar irradiance), as shown in Fig. 4c. The output from the model is the forecasted solar irradiance for the upcoming day. Additionally, as illustrated in Fig. 4d, the model of wind speed forecasting is similar to the solar irradiance-based forecasting.

The effectiveness of the ANN-based forecasting model is confirmed by comparing it with LR and SVM. Root mean square error (RMSE) and mean absolute percentage error (MAPE) are two performance measures used in this study to assess prediction accuracy, which can be computed as follows:

15 $$ {\text{MAPE = }}\left( {\frac{{\left| {{\mathrm{Forecast}}{ - }{\mathrm{Real}}} \right|}}{{{\mathrm{Real}}}}} \right) \times 100. $$

16 $$ {\text{RMSE = }}\sqrt {\frac{{\sum\nolimits_{{\text{t = 1}}}^{{\text{t = 24}}} {\left( {{\mathrm{Forecast}}{ - }{\mathrm{Real}}} \right)^{{2}} } }}{24}} . $$

Although MAPE is a popular measure of predictive accuracy, it becomes ill-defined for solar irradiance data due to zero values during nighttime periods. As a result, the MAPE is not computed for the solar irradiation forecast.

The NGs suggested in this work are in Zaafarana, Egypt, specifically along the western coast of the Gulf of Suez^30^. The forecasting models are trained and tested using historical data from NASA Power for weather conditions, including solar irradiation, temperature, and wind speed^31^, and from a previous study for load and price^25^.

#### Demand side management (phase II)

There are two categories for electrical loads, controllable and uncontrollable loads. Uncontrollable loads, such as microwaves, TVs, and laptops have limited scheduling flexibility compared to controllable loads, such as washing machines, electric water heaters, and other appliances that can adjust their operating times^13^. Approximately 20–30% of all connected loads are actively controlled. In this study, to reduce energy usage, these loads can be moved from one time interval to another using the day-ahead pricing profile^8,32^.

The proposed load-shifting scheme considers appliance priority and predefined allowable shifting periods. Essential loads remain fixed, whereas flexible loads are shifted only within their allowable operating windows. Furthermore, the optimization includes constraints that prevent excessive load concentration after shifting, thereby avoiding the creation of new peak demand periods while maintaining customer comfort.

#### Day‑ahead scheduling of generation units (phase III)

Day-ahead forecasting of load demand, grid tariff, and PV and WT output power is used to plan the power of the sources in the NGs for the upcoming day. A novel bio-inspired metaheuristic algorithm, GOA, is employed to determine the optimal set points of the diesel generators and the batteries in the NGs.

- Goat optimization algorithm

The GOA is a recently developed bio-inspired metaheuristic that draws inspiration from the adaptive actions of goats. GOA is made to successfully balance exploration and exploitation by taking inspiration from their movement patterns, foraging techniques, and capacity to avoid parasites. An adaptive foraging method for global search, a directed movement technique for solution refinement, and a jump mechanism to escape local optima are the three main processes included in the algorithm^33^. The stages below provide a summary of the GOA’s mathematical model:

- Population initialization

Let *N* be the population size of goats in the search space and *Xi* let be a *d*-dimensional vector that represents each goat:

17 $$ X_{i} = \left[ {x_{i1} + x_{i2} + .... + x_{id} } \right]. $$

The number of decision variables is denoted by *d*. The initial population of goats is generated at random inside the designated lower and upper borders *LB* and *UB* of the search space:

18 $$ X_{i} = LB + (UB - LB) \times rand(d) $$

A *d*-dimensional vector of random values in the interval [0,1] is produced by the function rand (*d*).

(b) Exploration phase

Each goat explores the search space during this phase by moving randomly in different directions during this phase, inspired by its foraging behavior. The new location of each goat is updated using:

19 $$ X_{{_{i} }}^{t + 1} = X_{{_{i} }}^{t} + (UB - LB) \times R \times \alpha $$

where: *R* is a random variable (0,1), α is the exploration coefficient, and *X*_*i*_^*t*^ is the position of goat *i* at iteration *t*.

(c) Exploitation phase

To refine solutions, goats mimic goats’ tendency to travel toward optimal grazing locations by progressively approaching the best solution thus far. The exploitation phase is defined as:

20 $$ X_{{_{i} }}^{t + 1} = X_{{_{i} }}^{t} + (X_{{_{best} }}^{t} - X_{{_{i} }}^{t} ) \times \beta $$

where: *X*^*t*^_*best*_ is the best-performing goat*,* and *β* is the exploitation coefficient.

(d) Jump strategy for escaping local optima

One of GOA’s primary characteristics is its ability to escape local optima through abrupt leaps, inspired by goats’ exceptional mobility in rugged environments. Here is a mathematical representation of this:

21 $$ X_{{_{i} }}^{t + 1} = X_{{_{i} }}^{t} + (X_{{_{r} }}^{t} - X_{{_{i} }}^{t} ) \times \gamma $$

where: γ is the jump coefficient and *X*^*t*^_*r*_ is a randomly selected goat from the population.

(e) Parasite avoidance and solution filtering

Goats instinctively stay away from parasite-infested areas to preserve the solution’s quality. By eliminating ineffective solutions and developing new ones, GOA replicates this strategy. A population’s position is reset when its fitness drops to less than 20% using:

22 $$ X_{i}^{t + 1} = LB + (UB - LB) \times rand(d) $$

(f) Stopping criteria

**Fig. 5:**
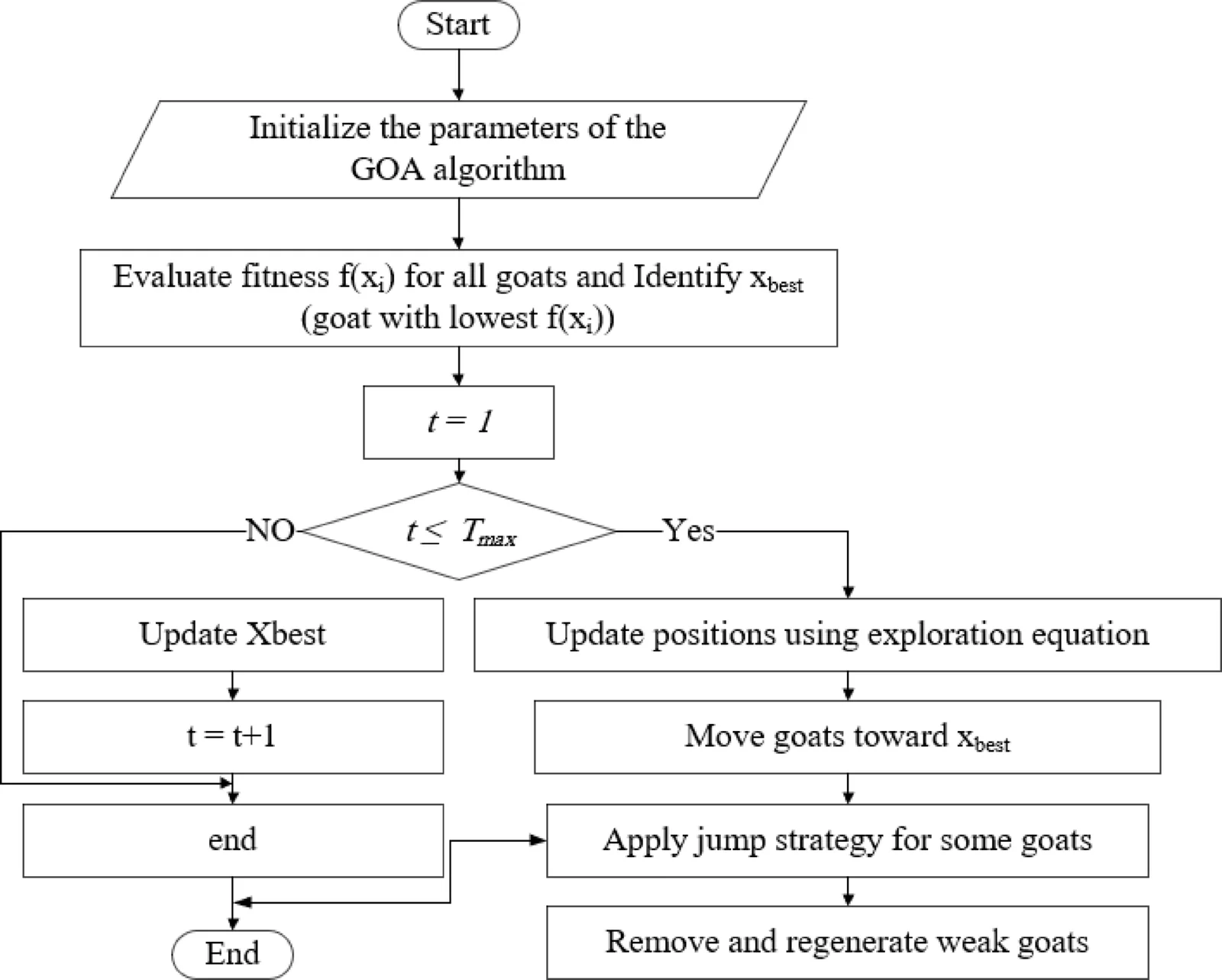
Flowchart of GOA.

When one of these conditions is satisfied, GOA ends: first, the maximum iterations (*T*_*max*_) are reached, and second, fitness improvement is less than a predetermined threshold. Figure 5 shows the flowchart that provides the main GOA procedures.

### Level II: real‑time scheduling

To fully exploit the advantages of multi-NGs, sophisticated control approaches are necessary for power sharing, fault tolerance, energy management, disturbance adaptation, and effective uncertainty management. This implies that there are many papers use MPC in managing MNGs. The capabilities of MPC encompass predicting future disturbances, enhancing system performance across a forecast horizon, and employing real-time feedback for adaptive control. MPC improves economic efficiency by accounting for the unpredictability of renewable energy sources, demand load variations, and fluctuations in electricity market tariffs^34,35^. The MPC formulation is based on the same objective function and system constraints employed in the day-ahead scheduling level; however, it is adapted for real-time operation with a finer time resolution (15-min intervals instead of hourly) and implemented in a receding-horizon framework.

**Fig. 6:**
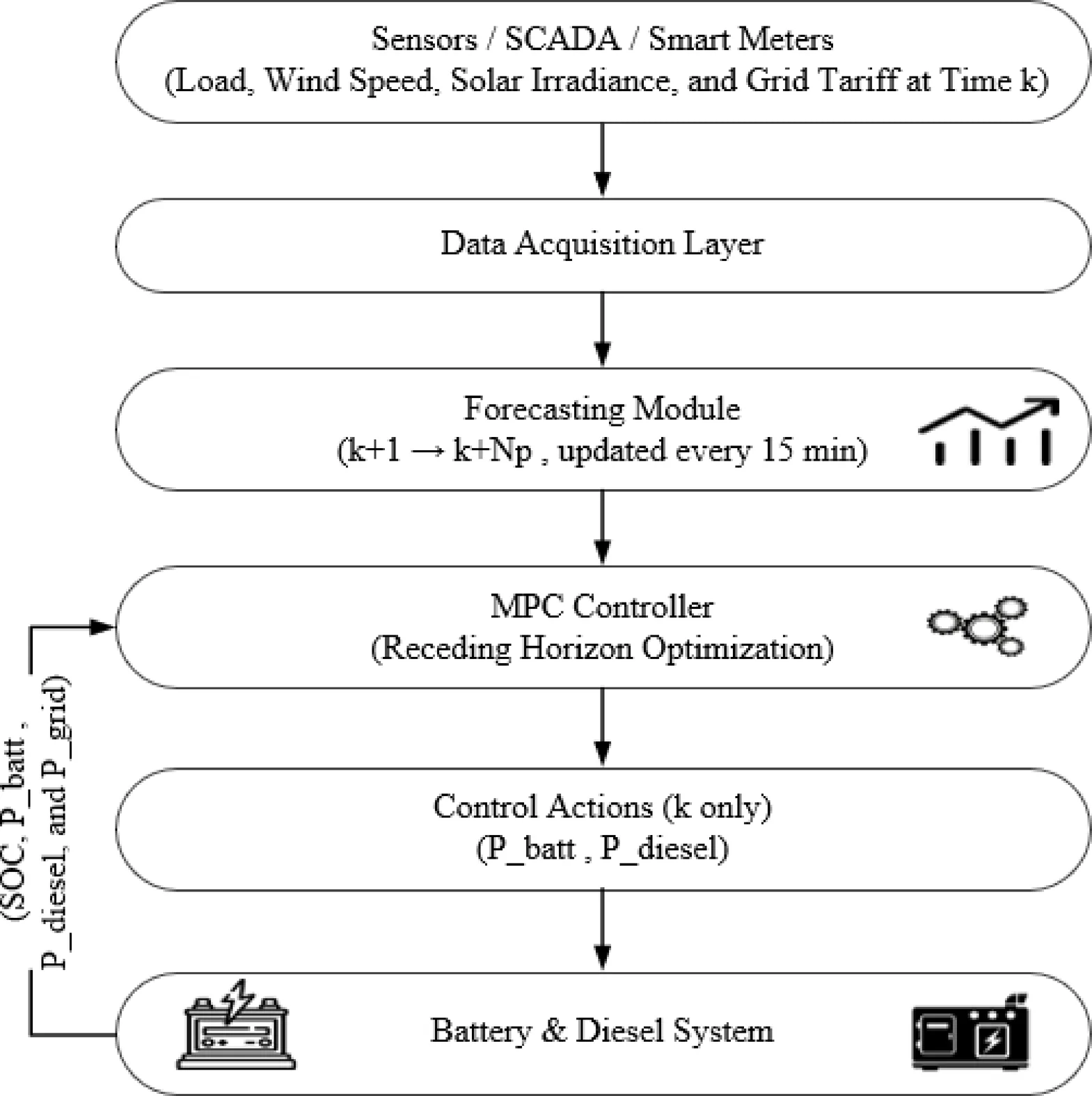
Real-time energy scheduling framework based on MPC.

To optimally coordinate distributed energy resources under dynamic operating conditions, a real-time EMS employing MPC that updates the set points of the generation units during real-time operation is recommended to achieve economical operation^36,37^. Uncertainty is not explicitly embedded via stochastic or robust optimization at the day-ahead stage. Instead, the day-ahead optimization relies on deterministic point forecasts generated by the ANN model for load demand, electricity prices, and weather conditions. During real-time operation, uncertainties are handled through the MPC framework, which continuously updates the optimization using actual measurements and rolling forecasts. Consequently, forecast errors are compensated through repeated optimization at each sampling interval. Only the first control action is implemented to the model, and the optimization is repeated at the upcoming time step in a receding horizon manner. This strategy enables adaptive and flexible real-time decision-making, enhances economic performance, ensures constraint satisfaction, and improves system reliability under renewable intermittency and load uncertainty^2,38,39^. Figure 6 provides the flowchart for the suggested real-time EMS for economical dispatch.

## Simulation results

The efficacy of the proposed EMS in decreasing daily operational costs is investigated in this section.

### Day‑ahead scheduling (level I) results

The generating units of the NGs are scheduled based on the weather, price, and load demand forecasts for the coming day.

#### Day‑ahead forecasting

**Fig. 7:**
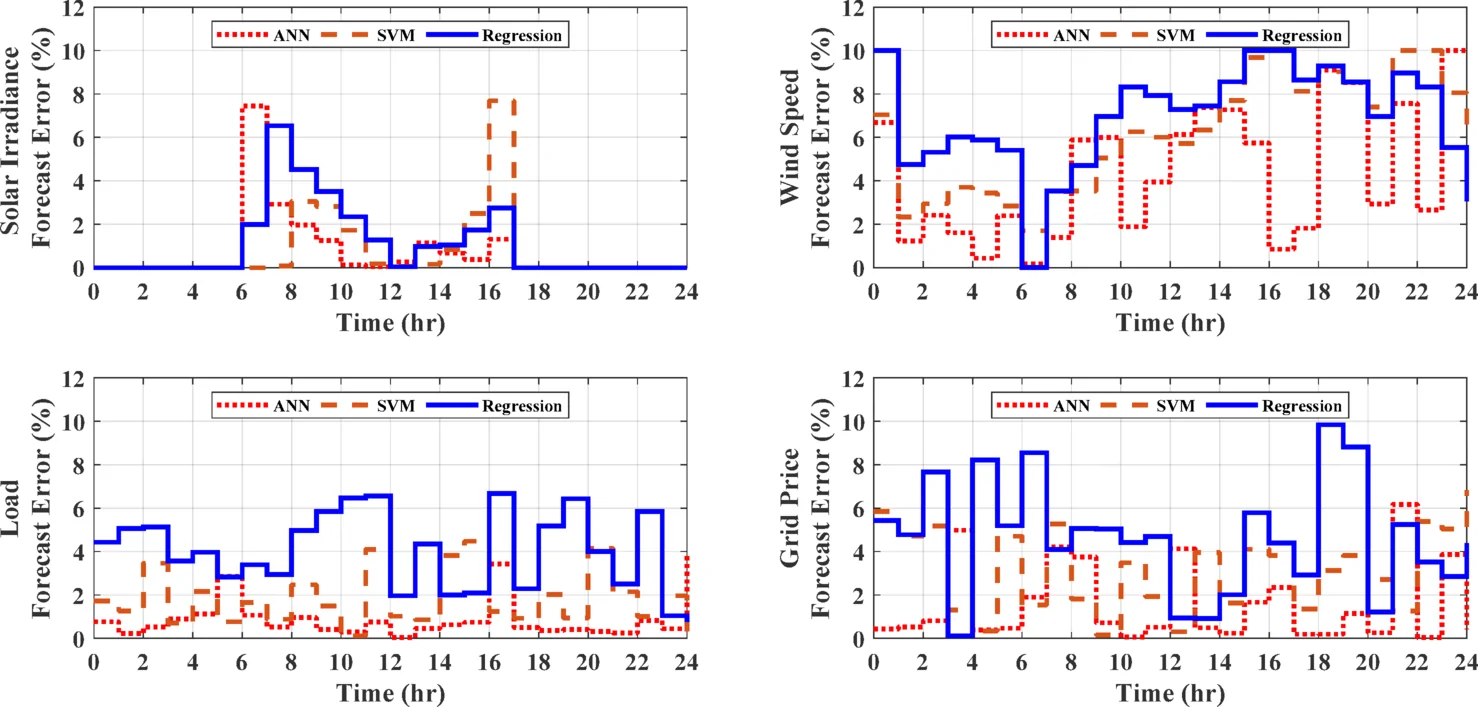
Forecast error for solar irradiance, wind speed, demand load, and grid price.

For LR, SVM, and ANN-based forecasting, the RMSE are 8.86 W/m^2^, 6.86 W/m^2^, and 3.70 W/m^2^, respectively. The MAPEs for load, grid tariff, and wind speed forecasting using linear regression are 4.01%, 4.64%, and 6.86%, respectively. In contrast, the MAPEs for load, grid tariff, and wind speed forecasting using SVM-based methods are 1.82%, 3.18%, and 6.21%, respectively. However, the MAPEs for load, grid tariff, and wind speed forecasting using ANN-based methods are 0.90%, 1.60%, and 4.56%, respectively. As seen in Fig. 7, ANN offers lower predictive error for load, grid tariff, solar irradiation, and wind speed than SVM and conventional LR methods. As indicated in Table 2, it can be said that, when MAPE and RMSE are considered, LR-based and SVM-based methods are less accurate than ANN-based short-term forecasting.

**Table 2:** Comparing the effectiveness of short-term forecasting techniques.

	ANN	SVM	LR
RMSE (W/m^2^) of solar irradiance	3.70	6.86	8.86
RMSE (m/s) of wind speed	0.30	0.43	0.64
RMSE ($/kWh) of grid tariff	0.005	0.008	0.0142
RMSE (kW) of load	0.13	0.22	0.56
MAPE (%) of load	0.90	1.82	4.01
MAPE (%) of grid tariff	1.60	3.18	4.64
MAPE (%) of wind speed	4.56	6.21	6.86

#### Day‑ahead scheduling of generation units

**Fig. 8:**
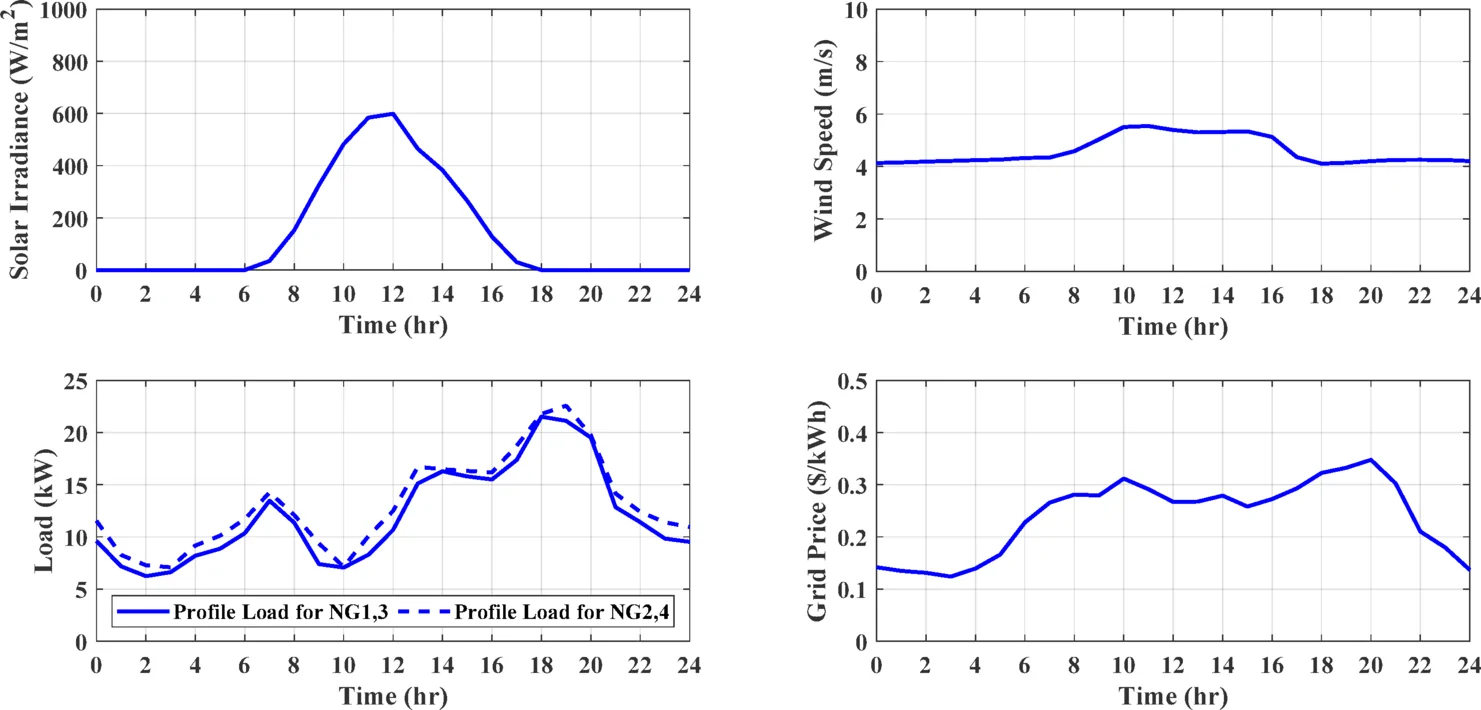
Forecasting data of grid tariff, load, wind speed, and solar irradiance.

The predicted loads, surrounding air temperature, solar intensity, wind speed, and grid price for Zaafarana City, Egypt, for the upcoming day are shown in Fig. 8. To assess the GOA technique operational costs and user comfort, various cases are compared with those of PSO, HHO, and DOA algorithms.

- Scenario I: individual operation of NGs

In this study, each NG works independently from the adjacent grids. NG1 and NG3 have typical configurations, as do NG2 and NG4. This study uses four metaheuristic techniques to obtain the operating points of the diesel generator and the battery.

**Fig. 9:**
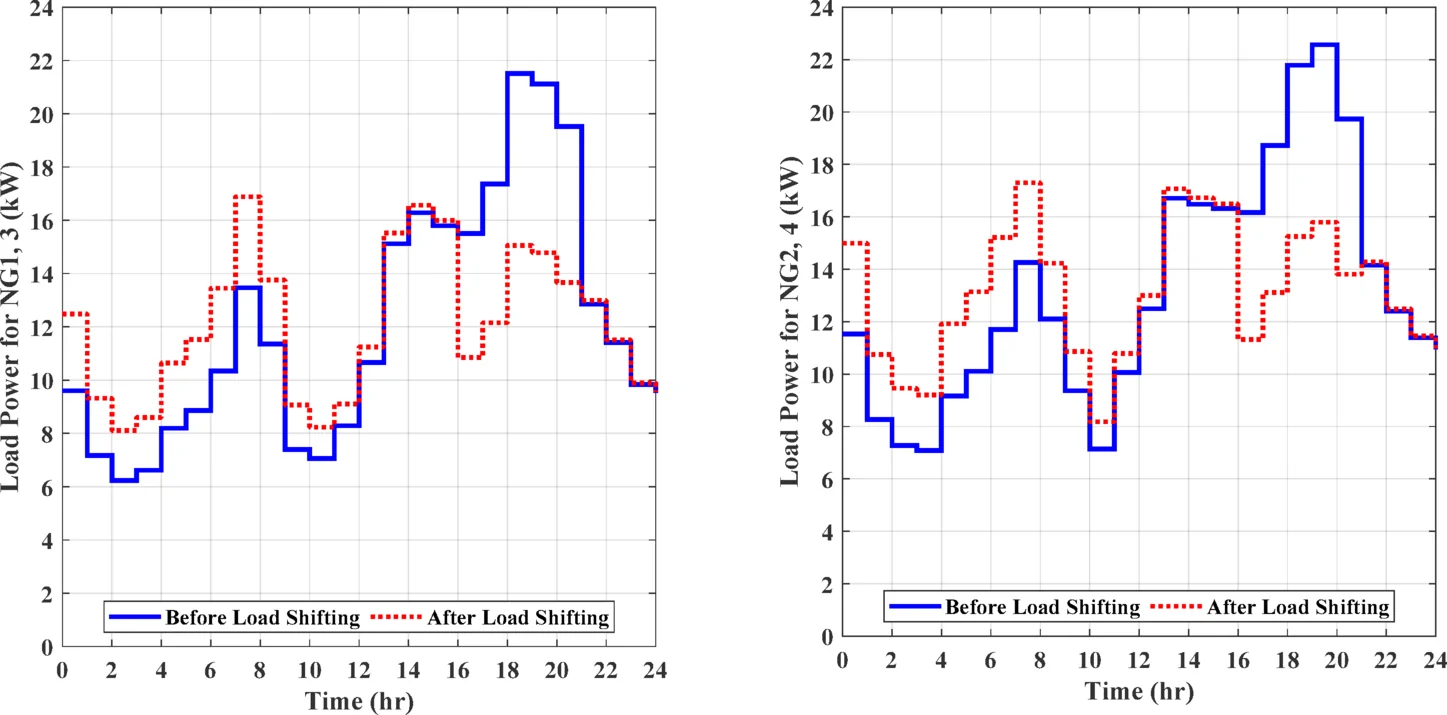
Daily load curve for NGs.

**Fig. 10:**
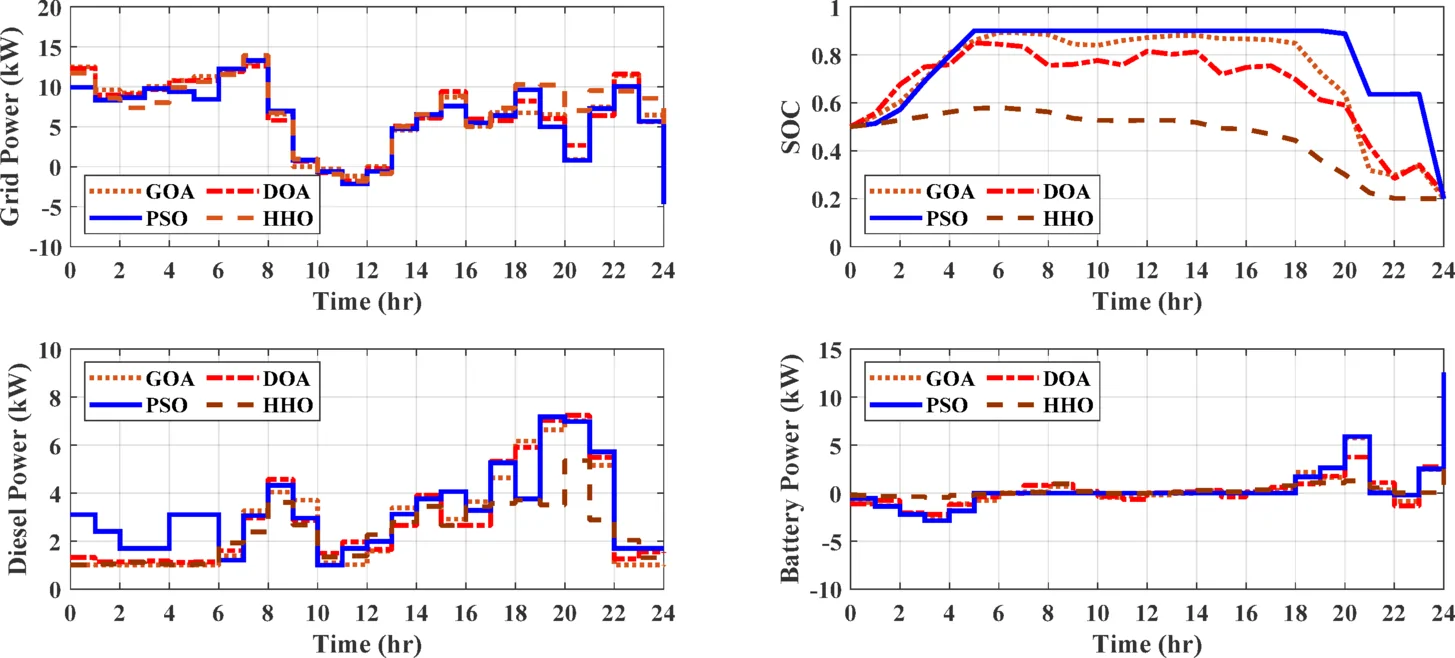
Power components and SOC for the NG1 during scenario I with DSM.

**Fig. 11:**
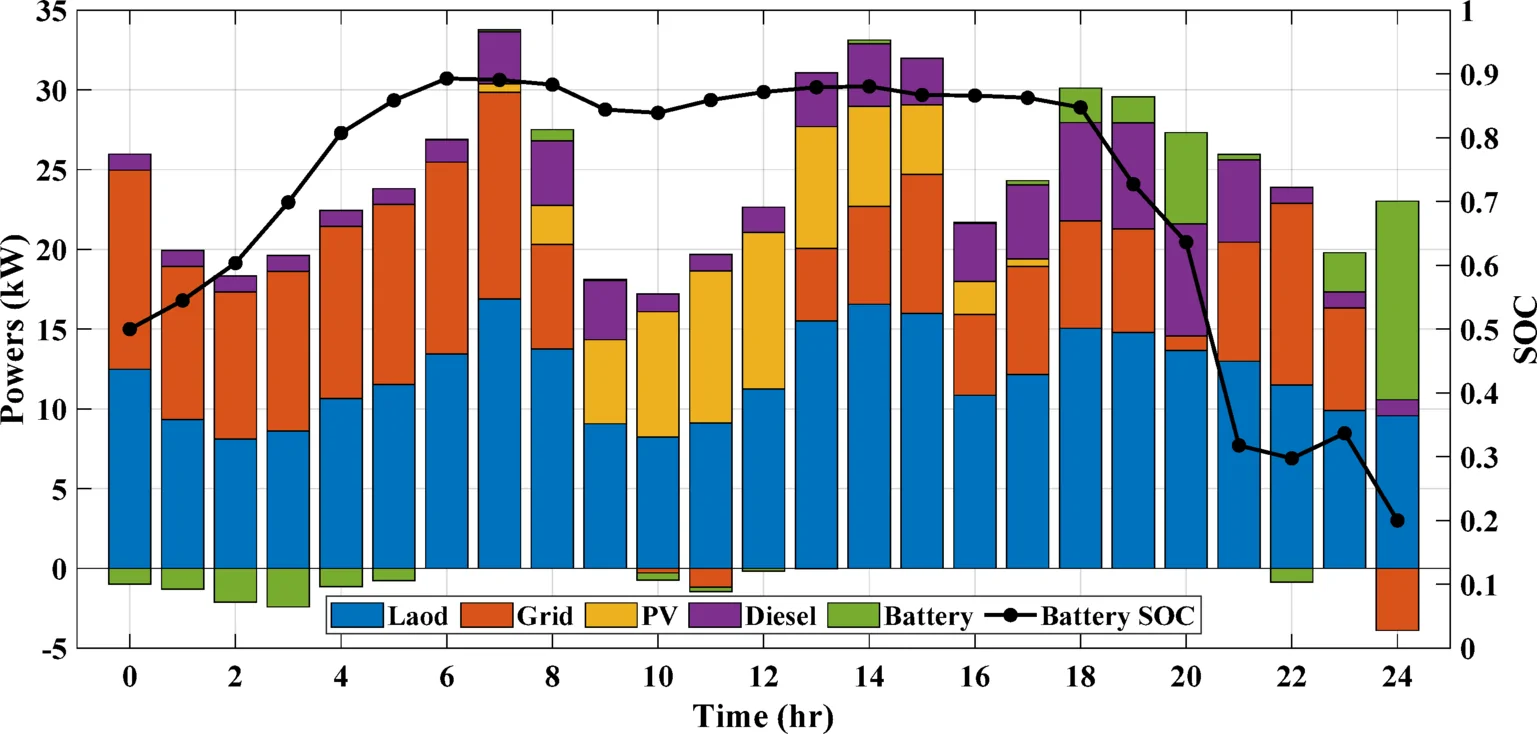
Stacking of power production and SOC based on GOA for NG1, scenario I with DSM.

For NG1, the total operating cost per day calculated by GOA is approximately $64.55, whereas DOA reports $65.37, HHO shows $66.37, and PSO indicates $66.43 without applying DSM. To minimize daily operating costs, a DSM technique known as load shifting is utilized. As illustrated in Fig. 9, this method moves controllable loads from periods of high demand to periods of low production and cost, which take place between roughly 4 p.m. to 8 p.m. As a result, the overall operating cost minimized to $61.02 by GOA, $61.89 by DOA, $62.61 by HHO, and $63.09 by PSO. As seen in Table 3, implementing DSM minimizes peak load and increases the load factor. The day-ahead optimal setpoints of NG1 sources generated by GOA, DOA, HHO, and PSO are illustrated in Fig. 10. The optimal stacking of power production and SOC based on GOA for NG1 are shown in Fig. 11. At 2 a.m., the load at NG1 (8.08 kW) is optimally supplied by different sources. The battery takes 2.14, 2.06, 0.36, and 2.23 kW (charging mode), while the diesel generator produces 1.00, 1.14, 1.11, and 1.70 kW, and the grid supplies 9.22, 9.03, 7.35, and 8.63 kW under the GOA, DOA, HHO, and PSO algorithms, and the PV produces 0 kW, respectively. Similarly, at 8 p.m., the load at NG1 (13.66 kW) is met by 5.74, 3.75, 1.28, and 5.89 kW from the battery (discharging mode), 7.01, 7.24, 5.36, and 6.99 kW from the diesel generator, and 0.92, 2.67, 7.03, and 0.78 kW from the grid for GOA, DOA, HHO, and PSO, respectively, and 0 kW from the PV, as demonstrated in Figs. 10 and 11.

**Table 3:** The summary of the daily load curves before and subsequent DSM.

	Peak Load (kW)		Peak load reduction (%)		Load factor	
	Before	After	Before	After	Before	After
NGs (1,3)	21.52	16.89	–	21.51	0.56	0.71
NGs (2,4)	22.56	17.30	–	23.31	0.58	0.76
MNGs	87.37	68.38	–	21.74	0.58	0.74

**Fig. 12:**
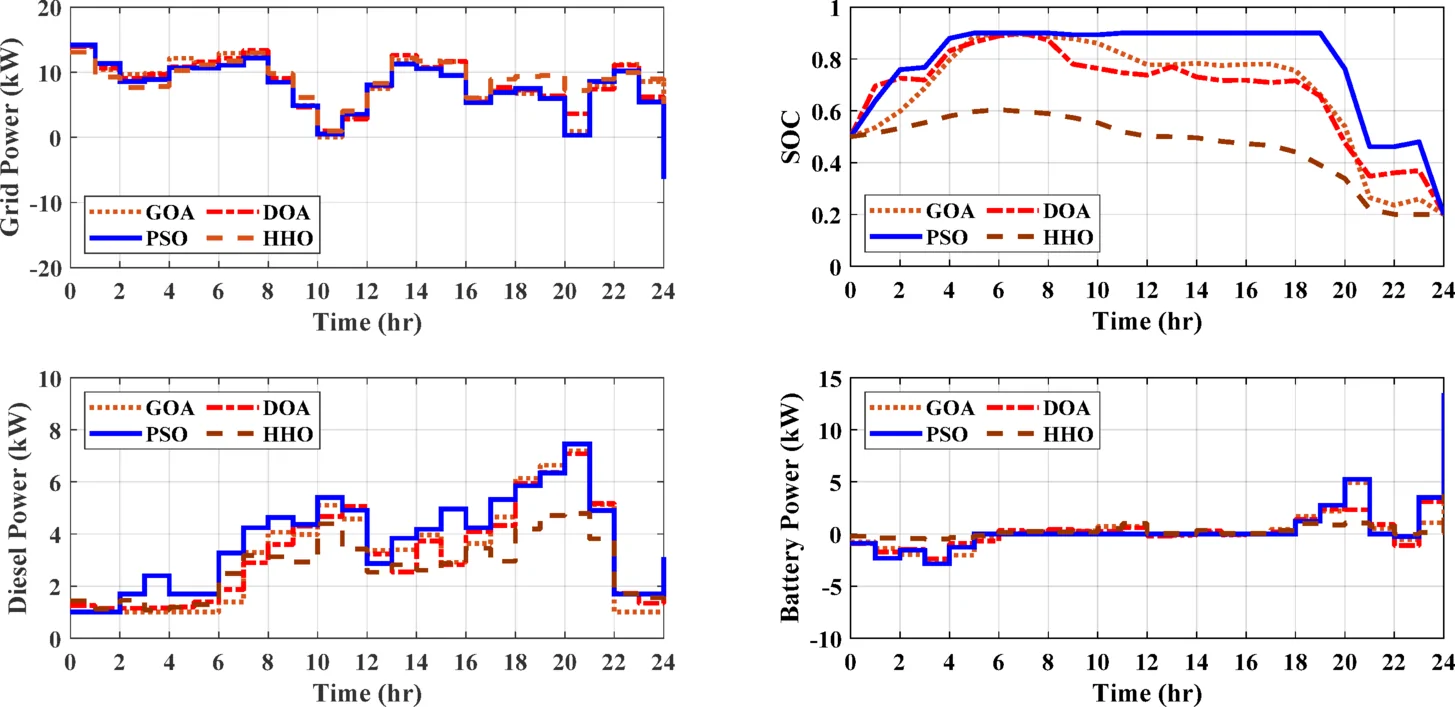
Power components and SOC for the NG2, scenario I with DSM.

**Fig. 13:**
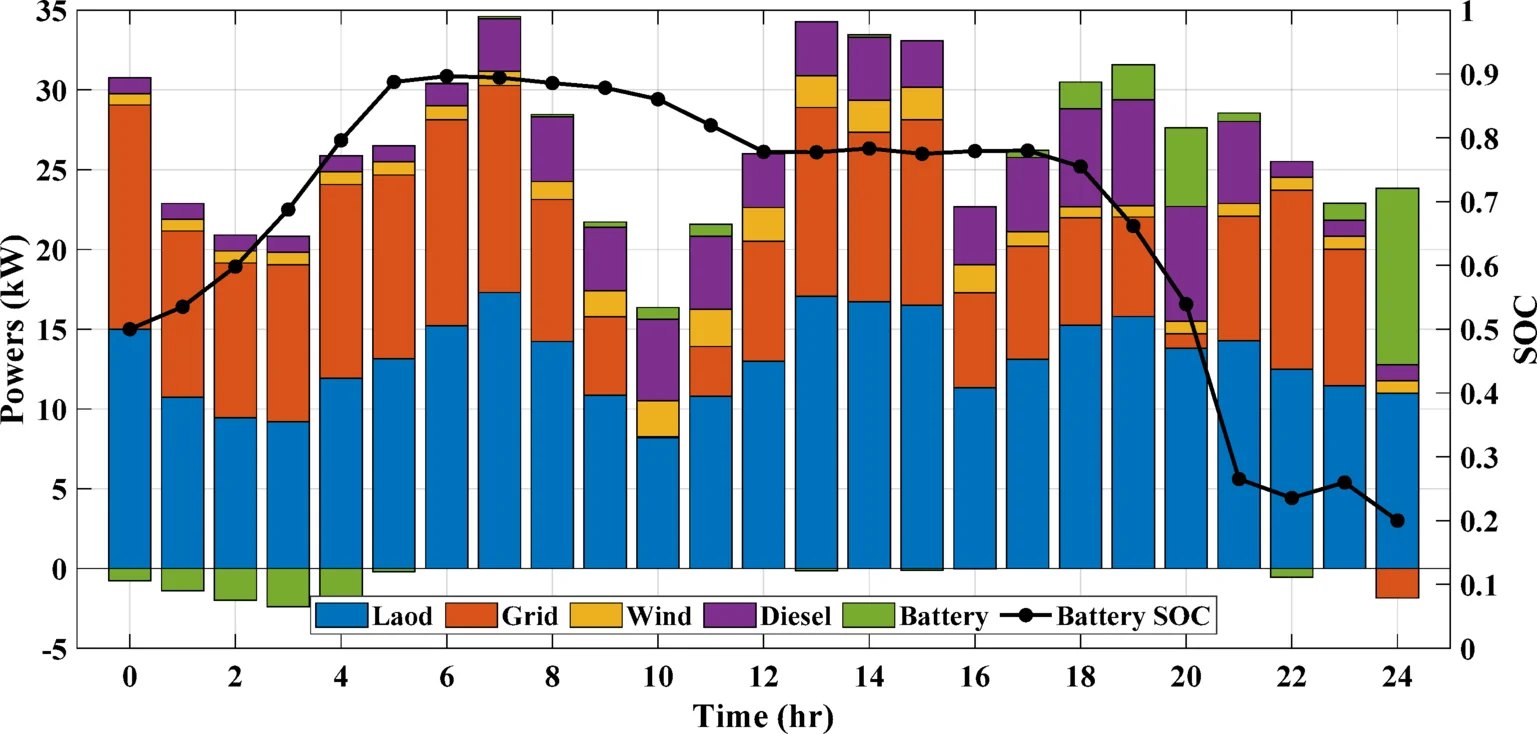
Stacking of power production and SOC based on GOA for NG2, scenario I with DSM.

For NG2, the overall operating cost per day determined by GOA, is roughly $78.14, but DOA reports $78.99, HHO indicates $79.44, and PSO shows $79.67 without using DSM, and with applying DSM technique, as indicated in Fig. 9. Consequently, the operating cost per day is decreased to $74.60 by GOA, $75.41 by DOA, $75.94 by HHO, and $76.16 by PSO. The day-ahead optimal setpoints of NG2 sources investigated by GOA, DOA, HHO, and PSO are depicted in Fig. 12. The optimal stacking of power production and SOC based on GOA for NG2 are observed in Fig. 13. At 3 a.m., the load at NG2 (9.20 kW) is optimally supplied by various sources. The battery stores 2.41, 2.37, 0.47, and 2.85 kW, while the diesel generator produces 1.00, 1.17, 1.30, and 2.4 kW, and the grid provides 9.84, 9.63, 7.82, and 8.88 kW under the GOA, DOA, HHO, and PSO algorithms, respectively, and the WT produces 0.78 kW. Also, at 2 p.m., the load at NG2 (16.73 kW) is satisfied by 0, 0, 0.36, and 0 kW from the battery, 3.96, 3.73, 2.61, and 4.18 kW from the diesel generator, and 10.61, 10.71, 11.75, and 10.54 kW from the grid for GOA, DOA, HHO, and PSO, respectively, and 2.1 kW from the WT, as displayed in Figs. 12 and 13.

A sensitivity analysis was performed on the population size and maximum iterations. Multiple simulations were conducted, varying the population size from 60 to 140 and iterations from 2000 to 6000. As illustrated in Table 4, the results show that a population size of 120 and 5000 iterations yield the most stable and optimal solution. A thorough comparison with alternative algorithms was carried out to confirm the efficacy and resilience of the suggested GOA-based EMS, as shown in Tables 5, 6, 7, and 8. Indicators of statistics (mean, best, worst, and standard deviation) were used to conduct the evaluation over several independent runs. Additionally, the computational time analysis and the importance of the performance variations statistically was evaluated using the Wilcoxon signed-rank test.

**Table 4:** Sensitivity analysis of GOA hyperparameters.

Sensitivity analysis of GOA hyperparameters (population size and maximum iterations)			
Operating Cost ($) For NG1 with DSM			
[60, 5000]	62.08	[120, 2000]	61.16
[80, 5000]	61.18	[120, 3000]	61.06
[100, 5000]	60.97	[120, 4000]	61.00
[120, 5000]	60.02	[120, 5000]	60.04
[140, 5000]	60.03	[120, 6000]	60.06

**Table 5:** Statistical outcomes over ten runs for NG1 and NG2 without DSM.

	Operating Cost ($), Scenario I without DSM				
	Best	Mean	Worst	Std	Improvement over GOA (%)
For NG1					
DOA	65.20	65.37	65.71	0.16	1.25
HHO	66.37	66.37	66.70	0.17	2.74
PSO	64.44	66.43	74.65	3.01	2.83
GOA	64.23	64.55	64.80	0.16	–
For NG2					
DOA	78.63	78.99	79.63	0.34	1.08
HHO	79.17	79.44	80.64	0.19	1.64
PSO	77.87	79.67	82.59	1.49	1.92
GOA	77.97	78.14	78.54	0.17	–

**Table 6:** Statistical outcomes over ten runs for NG1 and NG2 with DSM.

	Operating Cost ($), Scenario I with DSM				
	Best	Mean	Worst	Std	Improvement over GOA (%)
For NG1					
DOA	61.65	61.89	62.13	0.16	1.41
HHO	62.46	62.61	63.15	0.19	2.54
PSO	61.57	63.09	64.76	0.95	3.28
GOA	60.87	61.02	61.17	0.11	–
For NG2					
DOA	75.19	75.41	75.87	0.24	1.07
HHO	75.81	75.94	76.25	0.13	1.76
PSO	74.95	76.16	77.44	0.75	2.05
GOA	74.48	74.60	74.72	0.08	–

**Table 7:** Computational time analysis.

Computational time for NG1 and NG2 (sec)								
	Without DSM				With DSM			
PSO	PSO	HHO	DOA	GOA	PSO	HHO	DOA	GOA
NG1	15	22	40	60	16	25	43	60
NG2	16	24	41	61	17	26	42	62

**Table 8:** Wilcoxon test over ten runs for NG1 and NG2.

	During Scenario 1 without DSM		During Scenario 1 with DSM	
	*p* value	Significance	*p* value	Significance
For NG1				
GOA vs PSO	0.027344	✓	0.001953	✓
GOA vs HHO	0.001953	✓	0.001953	✓
GOA vs DOA	0.001953	✓	0.001953	✓
For NG2				
GOA vs PSO	0.003906	✓	0.001953	✓
GOA vs HHO	0.001953	✓	0.001953	✓
GOA vs DOA	0.001953	✓	0.001953	✓

(b) Scenario II: operation of a grid-tied multi-NGs cluster

**Fig. 14:**
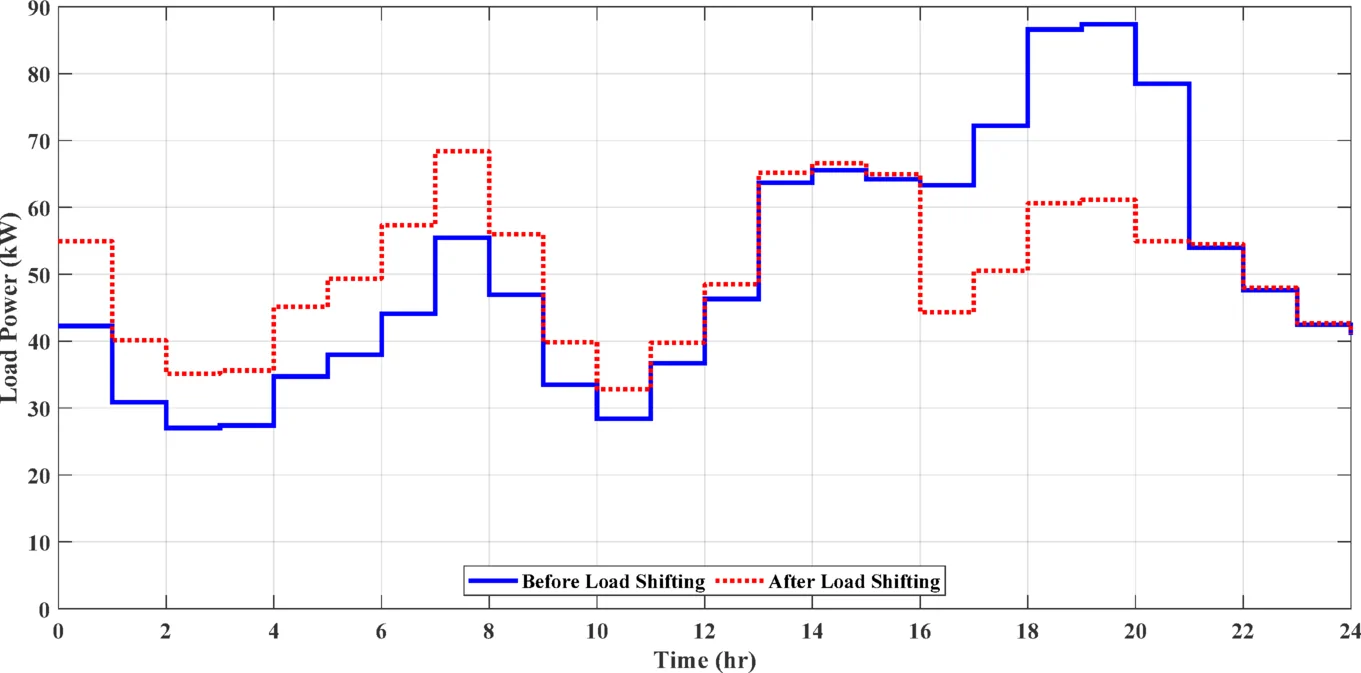
Daily load curve for MNGs.

**Fig. 15:**
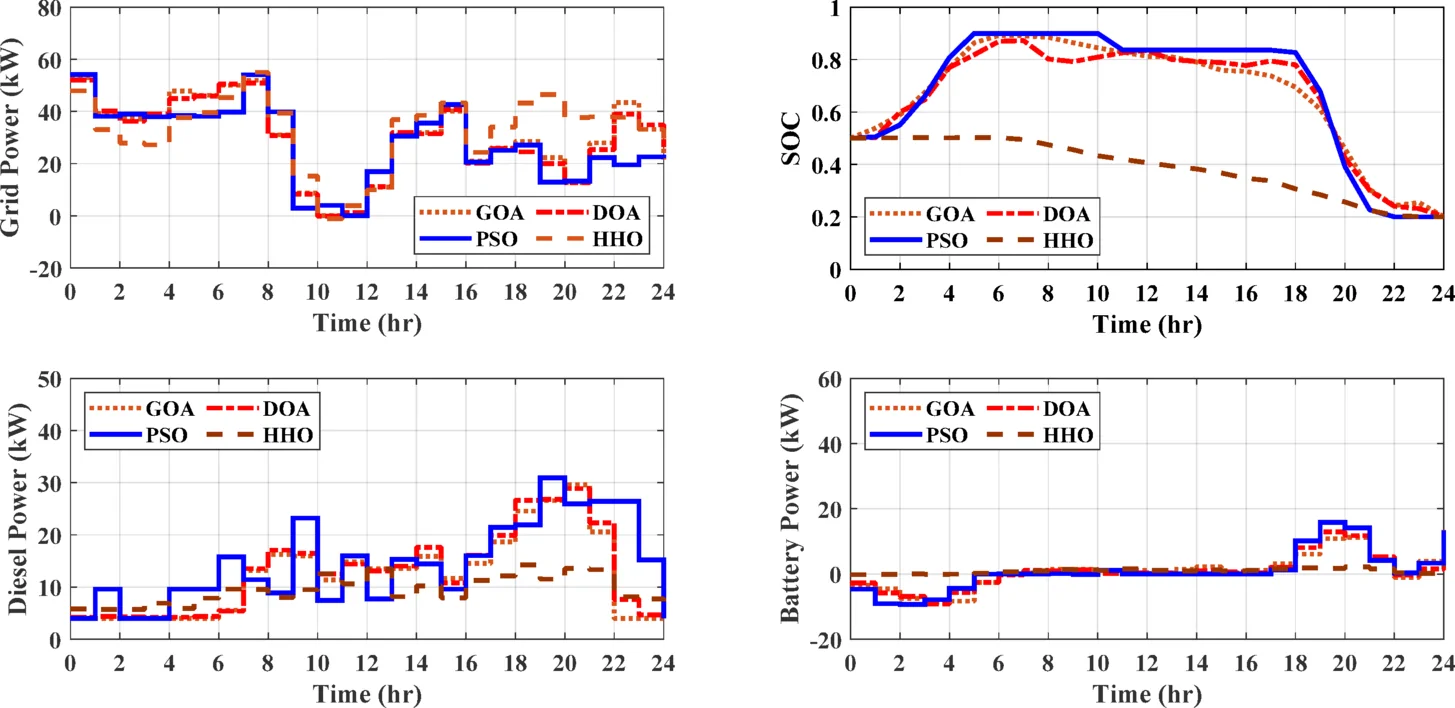
Power components and SOC for the MNGs, scenario II with DSM.

**Fig. 16:**
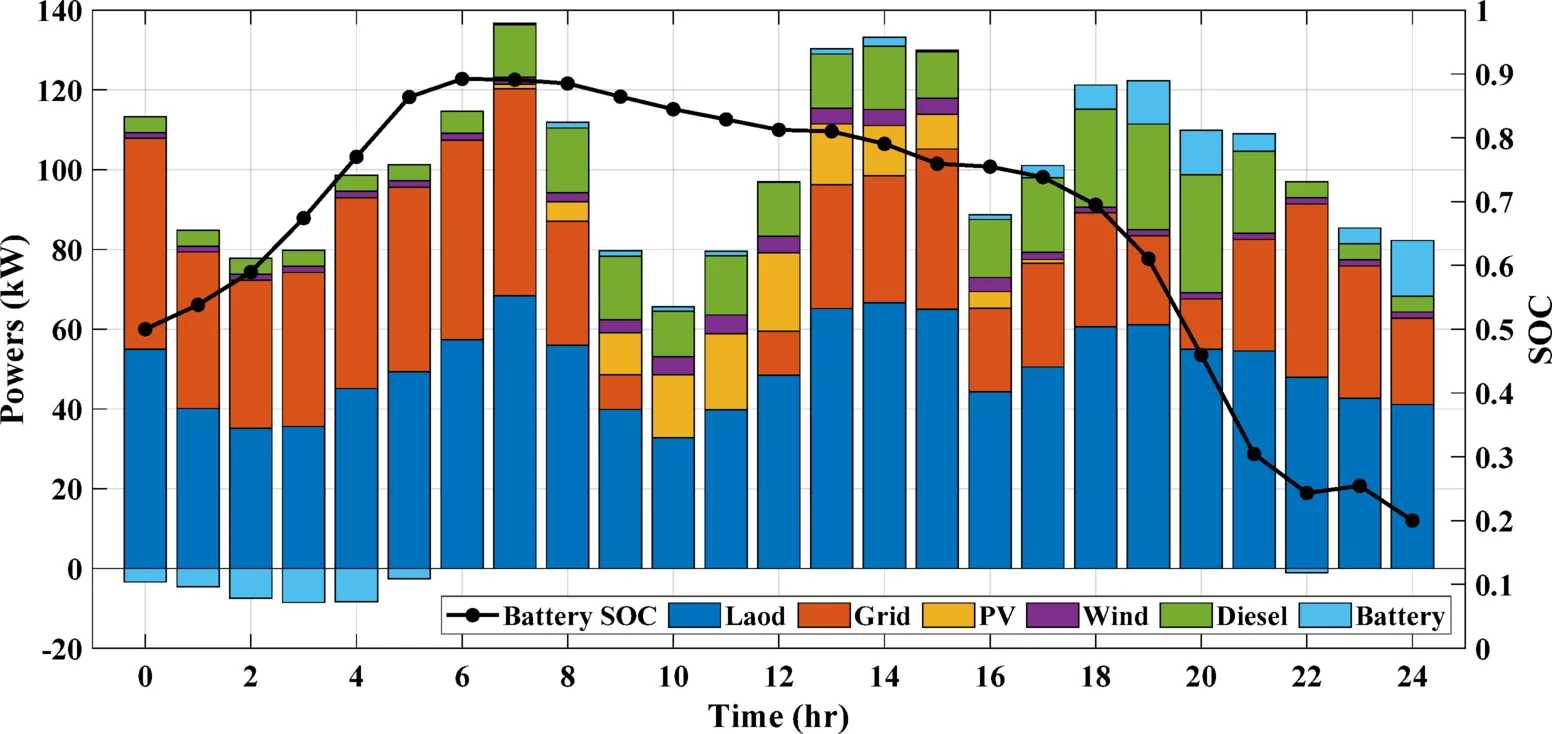
Stacking of power production and SOC based on GOA for MNGs, scenario II with DSM.

This case study assesses the performance of four NGs operating in grid-connected mode as a single microgrid in a cluster. Figure 14 illustrates the daily profile load, with peak demand of 68.38 kW at 7 a.m. and off-peak demand of 32.83 kW at 10 a.m., when implementing the DSM technique. GOA produces a daily operational cost of about $268.35 for MNGs, while DOA, HHO, and PSO report costs of $269.37, $273.90, and $275.32 with DSM, respectively. The day-ahead optimal setpoints of MNGs sources evaluated by GOA, DOA, HHO, and PSO are depicted in Fig. 15. The optimum stacking of power production and SOC based on GOA for MNGs are given in Fig. 16. At 7 a.m., the load at MNGs (68.38 kW) is optimally supplied by different sources. The battery gives 0, 1.11, 0.97, and 0 kW, while the diesel generator provides 13.15, 13.54, 9.5, and 11.42 kW, and the grid transfers 51.95, 50.85, 54.60, and 54.07 kW under the GOA, DOA, and PSO algorithms, and the PV and WT produce 1.10 and 1.78 kW, respectively. Additionally, at 5 p.m., the load at MNGs (50.53 kW), the battery absorbs 3.12, 2.12, 1.55, and 1.22 kW (charging mode), 18.63, 19.88, 12.14, and 21.42 kW from the diesel generator, and 26.00, 25.76, 34.07, and 25.12 kW from the grid for GOA, DOA, HHO, and PSO, respectively, and 0.96 kW, 1.81 kW from PV and WT, as investigated in Figs. 15 and 16.

The day-ahead energy consumption cost reduces by about $23.85 with GOA, $22.83 with DOA, $18.30 with HHO, and $16.88 with PSO, compared to the base scenario 1 without DSM. With a percentage of cost savings of around 8.16%, the GOA algorithm outperforms the DOA, HHO, and PSO algorithms in achieving optimal setpoints for the battery and the diesel generator using the DSM strategy while taking daily operating costs into consideration.

### Real‑time scheduling (level II) results

**Fig. 17:**
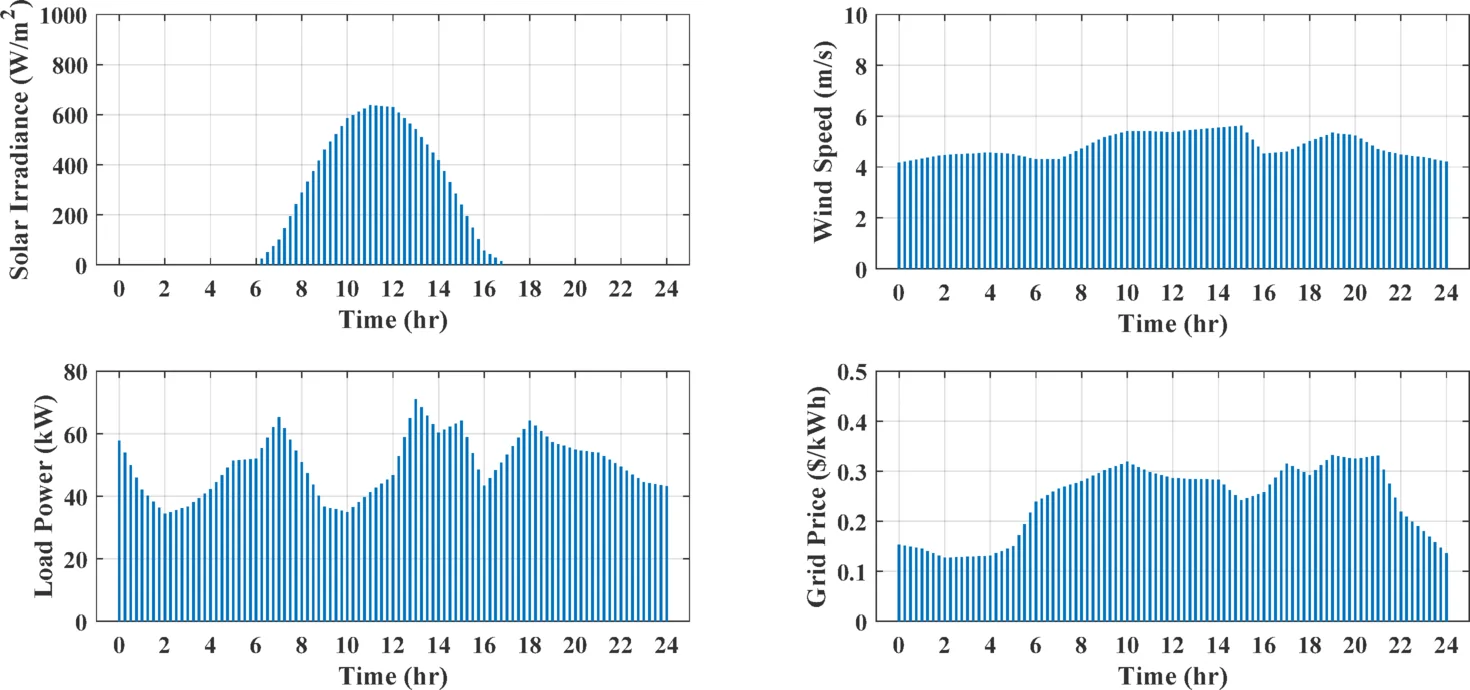
Actual grid price, solar irradiance, wind speed, and load demand data.

**Fig. 18:**
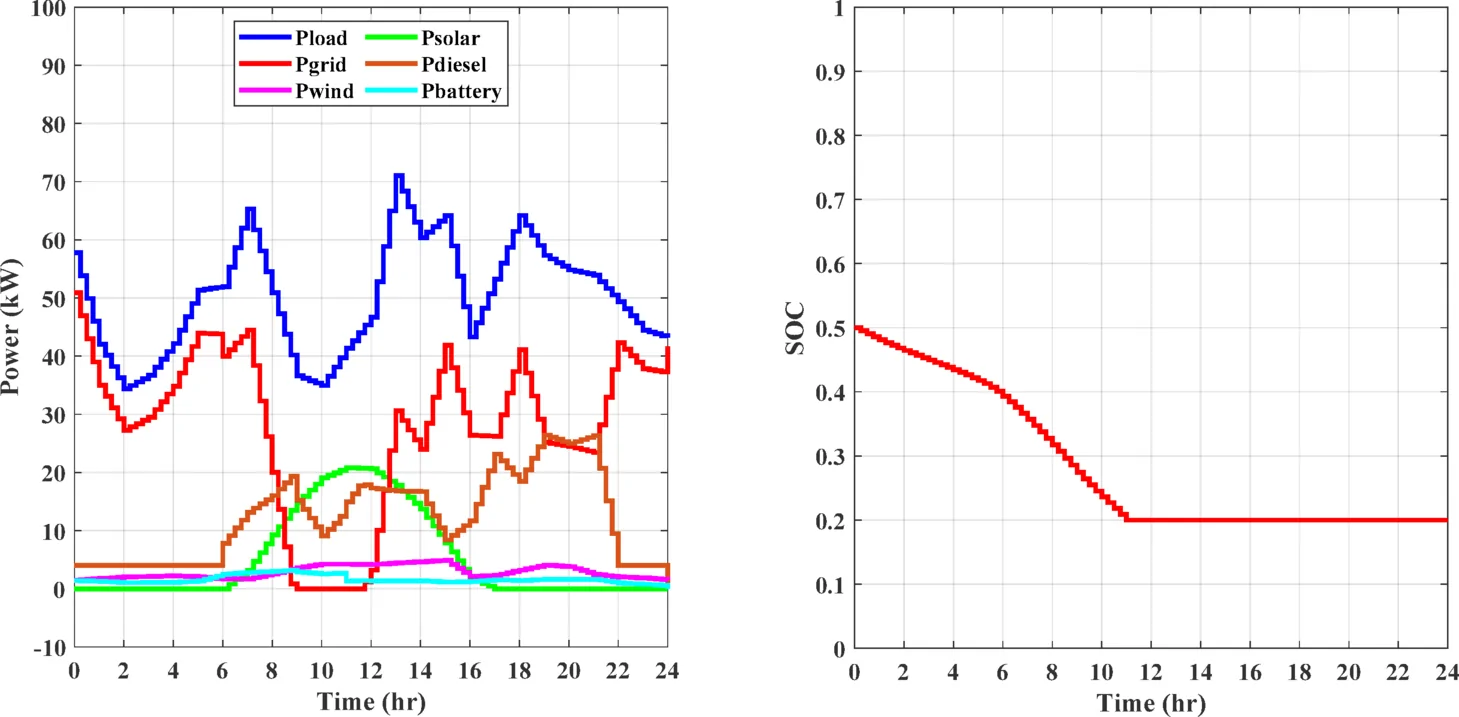
Power components and SOC for the MNGs using MPC.

**Fig. 19:**
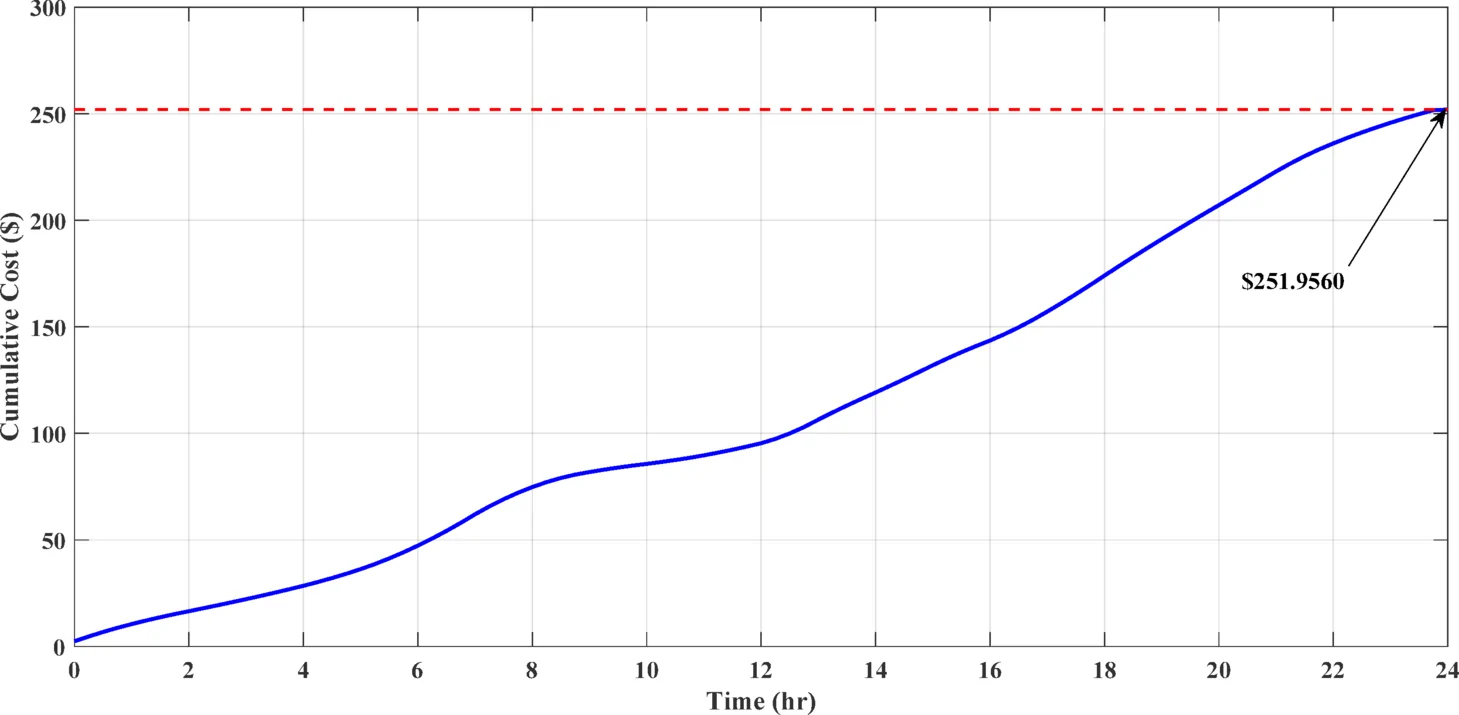
Cumulative cost for the MNGs using MPC.

**Fig. 20:**
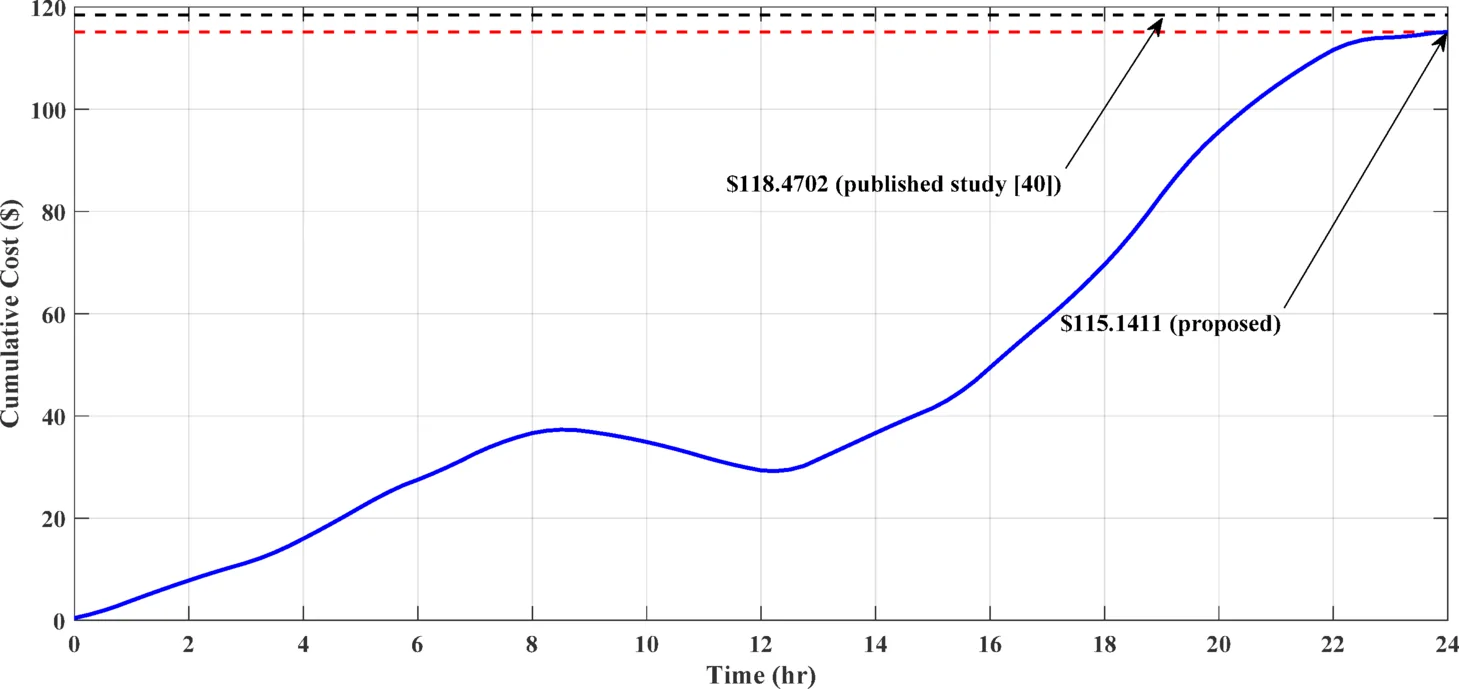
Cumulative cost for the MNGs using MPC compared to published study^40^.

Forecasting weather, electricity prices, and load demand always involves some degree of uncertainty. To effectively handle these uncertainties, a real-time EMS based on MPC is implemented, it uses day-ahead scheduling to continuously update and reschedule distributed energy resources’ operating setpoints., ensuring robust and cost-effective system operation. The controller continuously updates system states and forecasts, then determines optimal power dispatch for battery storage systems, diesel generators, and grid interaction within a moving prediction horizon. The objective function incorporates operational cost minimization, and grid power exchange penalties, while explicitly enforcing technical constraints such as SOC bounds, generator capacity limits, and power balance equations. In this study, real-time data is compared with projected data from scenario II, which was obtained through GOA. To maintain effective coordination between layers, the day-ahead GOA schedule serves as a reference trajectory rather than a rigid constraint for the real-time MPC layer. During real-time operation, the MPC updates control actions using updated short-term forecasts and actual system measurements. When significant deviations occur due to severe forecast errors or unforeseen disturbances, the MPC adjusts the operating strategy to prioritize real-time feasibility and cost-effectiveness over tracking the original day-ahead plan. The framework addresses communication and data latency; If the 15-min rescheduling time is far longer than the average communication delays in contemporary wiring or wireless intelligent grid systems, which vary from milliseconds to a few seconds. The actual solar irradiation, wind speed, load demand, and utility pricing statistics are displayed in Fig. 17. At 12 p.m., the load at MNGs (46.71 kW) is provided by 1.38 kW from the battery (20% SOC), 17.30 kW from the diesel generator, 3.21 kW from the grid, and 20.65 kW and 4.16 kW from PV and WT, as seen in Fig. 18. The cumulative cost for the MNGs using MPC for this study and the published study^40^, is depicted in Figs. 19 and 20.

Forecasting accuracy has a direct impact on the day-ahead scheduling decisions since the optimization relies on the forecasted photovoltaic generation, wind power, load demand, and electricity prices generated by the ANN model. To address this concern, a sensitivity analysis has been added by introducing ± 10% forecasting errors in renewable generation, load demand, and electricity prices. The analysis evaluates the impact of these forecasting errors on the total operating cost as well as the battery charging/discharging schedule and diesel generator dispatch, as shown in Table 9, the results demonstrate that forecasting errors increase the operating cost and lead to modifications in the battery and diesel schedules. However, the proposed MPC-based real-time energy management framework effectively mitigates these impacts by continuously updating the optimization using real-time measurements and rolling forecasts, thereby maintaining reliable and economical operation despite prediction inaccuracies.

**Table 9:** Sensitivity analysis of the proposed EMS under different forecasting error scenarios.

Scenario	Cost ($)	Battery charging (kWh)	Battery discharging (kWh)	Diesel energy (kWh)
Base	251.96	0	38.75	299.25
PV − 10%	264.7	62.76	35.67	301.47
Wind − 10%	261.50	62.29	37.16	298.24
Load + 10%	298.83	65.17	39.19	303.01
Price + 10%	283.15	63.86	38.17	381.20

Based on simulation results, Table 10 will examine the daily operational cost summary. The real-time EMS results in daily savings of $16.39, reducing operating costs by about 6.11% from $268.35 to $251.96.

**Table 10:** The operating costs based on the day-ahead and real-time scheduling.

	Individual NG Day-ahead daily cost ($)							
	Without DSM				With DSM			
	PSO	HHO	DOA	GOA	PSO	HHO	DOA	GOA
NG1	66.43	66.37	65.37	64.55	63.09	62.61	61.89	61.02
NG2	79.67	79.44	78.99	78.14	76.16	75.94	75.41	74.60
NG3	66.43	66.37	65.37	64.55	63.09	62.61	61.89	61.02
NG4	79.67	79.44	78.99	78.14	76.16	75.94	75.41	74.60
MNGs	292.20	291.62	288.72	285.38	278.502	277.10	274.60	271.24

MNGs Day-ahead daily operating cost ($) with DSM				MNGs Real-time daily operating cost ($) with DSM
PSO	HHO	DOA	GOA	MPC
275.32	273.90	269.37	268.35	251.96

## Conclusion

This paper proposed a comprehensive bi-level EMS for grid-connected multi-nanogrid systems, consisting of day-ahead and real-time scheduling layers to minimize the total operating cost while satisfying all technical constraints. In the first layer, an ANN-based forecasting model was employed to predict electricity prices, solar irradiance, wind speed, and load demand, achieving higher forecasting accuracy than support vector machine and linear regression models. The forecasted data were subsequently used by the GOA to determine the optimal day-ahead scheduling of diesel generators and battery energy storage systems. Comparative analysis with PSO, HHO, and DOA demonstrated the effectiveness of GOA in obtaining superior scheduling solutions. Furthermore, the proposed demand-side management strategy reduced the operating cost by approximately 4.92% through shifting controllable loads from high-price to low-price periods. For the coordinated operation of four interconnected nanogrids, the proposed framework achieved an additional operating cost reduction of 5.97% compared with independent operation without DSM.

In the second layer, the MPC strategy continuously updated the dispatch decisions based on real-time measurements, effectively compensating for forecasting errors in renewable generation, load demand, and electricity tariffs. As a result, the proposed real-time scheduling strategy achieved an additional daily operating cost reduction of approximately 4.04% while maintaining system power balance and battery state-of-charge constraints. Moreover, the sensitivity analysis demonstrated that the proposed EMS remained effective under forecasting uncertainties, with load forecasting errors having the greatest impact on operating cost, whereas the MPC successfully mitigated their influence through continuous corrective actions.

Future work will focus on incorporating electric vehicles, demand response programs, and extend the proposed EMS to larger interconnected multi-nanogrid systems with peer-to-peer energy trading.

## Data Availability

The datasets generated and/or analyzed during the current study are available from the corresponding author on reasonable request.

## Appendix

See Table 11.

**Table 11:** System, GOA, and MPC parameters.

System, GOA, and MPC parameters		
Photovoltaic	Wind turbine	Diesel generator
$$A=20 {m}^{2}$$	$${P}_{w}=5 kW$$	$${P}_{dg}=8 kW$$
$${\eta}_{pv}=18\mathrm{\%}$$	$${v}_{ci}=3 m/s$$	a = 0.03 $/h
$${\eta}_{conv}=94\mathrm{\%}$$	$${v}_{cf}=25 m/s$$	b = 0.2 $/kWh
$${N}_{pv}=5$$	$${v}_{r}=10.5 m/s$$	c = 0.01 $/kW^2^h
$${P}_{pv}=4 kW$$	$${N}_{w}=4$$	P_dg_ (min) = 1 kW
$${T}_{c}=25^\circ C$$	$${\eta}_{conv}=92\mathrm{\%}$$	P_dg_ (max) = 8 kW
		$${T}_{dg}^{up,down}$$ = 1 hr

Battery	
$${B}_{capacity}=20 kWh$$	$${N}_{cycle}=4000$$
CC_Bat_ = 300 $/kWh	$${SOC}_{min}=0.2$$
C_deg_ = 0.00025 $/kW	$${SOC}_{max}=$$ 0.9
$${B}_{batt}(min)=4 kWh$$	$${SOC}_{ini}=$$ 0.5
$${B}_{batt}(\mathrm{m}\mathrm{a}\mathrm{x})=18 kWh$$	$${\eta}_{ch}=0.9$$
*Δ T=1 hr*	$${\eta}_{dis}=0.9$$

GOA	
Maximum iterations (5000)	Number of delta goats per group (5)
Population size (120)	Maximum velocity (0.3)
Number of goat groups (4)	Initial exploration rate (0.9)
Number of alpha goats per group (2)	Initial exploitation rate (0.1)
Number of beta goats per group (3)	Learning factors (C1 = 2, C2 = 2, C3 = 1.5)

MPC
Sampling time (15 min)
Prediction horizon (24 h with 15-min intervals)
Control horizon (2 h)
